# The Burden and Trends of Breast Cancer From 1990 to 2017 at the Global, Regional, and National Levels: Results From the Global Burden of Disease Study 2017

**DOI:** 10.3389/fonc.2020.00650

**Published:** 2020-05-12

**Authors:** Peng Ji, Yue Gong, Ming-Liang Jin, Xin Hu, Gen-Hong Di, Zhi-Ming Shao

**Affiliations:** ^1^Key Laboratory of Breast Cancer in Shanghai, Department of Breast Surgery, Fudan University Shanghai Cancer Center, Fudan University, Shanghai, China; ^2^Department of Oncology, Shanghai Medical College, Fudan University, Shanghai, China; ^3^Institutes of Biomedical Science, Fudan University, Shanghai, China

**Keywords:** breast cancer, global burden of disease, burden trends, incidence, mortality

## Abstract

**Background:** Data on burden and changing trends of breast cancer are of value for policymaking. We aimed to determine the pattern of breast cancer incidence, mortality, and disability-adjusted life-years (DALYs), as well as temporal trends, from 1990 to 2017.

**Methods:** We collected detailed information on breast cancer between 1990 and 2017 using the results of the Global Burden of Disease study. The number of incident cases, deaths, and DALYs attributable to breast cancer are reported as well as age-standardized rates. Estimated annual percentage changes (EAPCs) in age-standardized rates were calculated to quantify the temporal trends. Moreover, the attributable burden to breast cancer risk factors was also estimated.

**Results:** There were 1,960,682 incident cases and 611,625 deaths of breast cancer globally in 2017, contributing to 17,708,600 DALYs. The age-standardized incidence rates (ASIRs) increased between 1990 and 2017, while the age-standardized mortality rates and DALY rates decreased. The corresponding EAPCs were 0.41, −0.62, and −0.56, respectively. These trends were heterogeneous across regions and countries. The increase in the ASIRs was more prominent in countries with a low sociodemographic index. The percentages of breast cancer deaths due to alcohol use and tobacco were decreasing, while deaths due to high body mass index and high fasting plasma glucose were increasing.

**Conclusion:** Breast cancer remained a major public health concern globally. The trends of incidence, mortality, and DALYs were heterogeneous across regions and countries, suggesting that the allocation of appropriate health care resources for breast cancer should be considered at the national scale and even at the subnational scale.

## Introduction

In women, breast cancer is the most frequently diagnosed cancer and the leading cause of cancer-associated death worldwide (1). Early breast cancer is considered a curable disease, while metastatic breast cancer is associated with poor outcomes. The common biological subtypes of breast cancer are luminal A, luminal B, human epidermal growth factor receptor 2 (HER2)-positive, and triple-negative breast cancer, according to the status of hormone receptors (estrogen and progesterone receptors) and expression of HER2 (2).

Despite the rapid development of diagnostic and therapeutic methods for breast cancer, different regions and individual countries develop at different speeds, leading to the substantial diversity of disease burden (3). Nonhereditary causes of breast cancer predominate but are still unclear; these causes include early menarche, late age at menopause, exogenous hormone intake, alcohol consumption, smoking, and obesity, all of which are commonly reported as risk factors (1). Hereditary causes account for only 5–10% of breast cancer cases, while germline mutations in breast cancer gene (BRCA)1 or BRCA2 account for 30% of inheritable breast cancer cases (4).

The prognosis of breast cancer patients is correlated with age at diagnosis, stage at diagnosis, pathological findings, treatments accepted, and regular follow-up (5). In terms of early detection, mammary screening can increase the proportion of early-stage breast cancer detection, despite controversy in its cost efficiency (3). Most high-income countries have promoted mammary screening programs for several years, while middle- and low-income countries are still in the initial stages (6). In terms of treatments, advances in endocrine therapies and targeted therapies over the past several decades have substantially improved the prognosis of hormone receptor-positive and HER2-positive patients, respectively. However, expensive targeted therapies, long-term endocrine therapies, and lifelong follow-ups undisputedly result in enormous pressure on individuals and the global healthcare system.

Summarizing and comparing breast cancer metrics and trends considering different aspects are important for decision making regarding health policies, screening protocols, and lifestyle guidelines, which might affect the outcomes of breast cancer patients and everybody's pattern of life.

The Global Burden of Disease (GBD) study provides annual estimated incidence, mortality, disability-adjusted life-years (DALYs) data made by age and sex, and temporal trends of breast cancer among different regions and countries. We collected GBD data concerning breast cancer from 1990 to 2017 and estimated the disease burden of breast cancer at the global, regional, and national levels. In addition, we presented the contributions of different risk factors to mortality due to breast cancer.

## Materials and Methods

### Study Data

We collected the annual incident cases, deaths, years lived with disability (YLDs), years of life lost (YLLs) and DALYs, and the age-standardized data of breast cancer from 1990 to 2017 from the Global Health Data Exchange query tool (http://ghdx.healthdata.org/gbd-results-tool) (7). The GBD 2017 study assessed the burden of breast cancer from a total of 195 countries and territories. In the GBD framework, the world was separated into seven super-regions and 21 regions in terms of geography. Moreover, the countries and territories were grouped based on their sociodemographic index (SDI) into five SDI quintiles: low, low-middle, middle, high-middle, and high (Supplementary Table 1). SDI, scaled from 0 to 1, is a summary measure of overall development based on the rankings of incomes per capita, average educational attainment, and fertility rates of all the areas in the GBD study. The methodological details of the GBD 2017 study have been described in previous studies (8–13).

### Estimation of Cancer Burden and Trends

We used the annual incident cases, deaths, YLDs, YLLs, and DALYs to estimate the burden of breast cancer. The Cause of Death Ensemble model was applied to estimate mortality based on the available data and covariates from the vital registration system and sample, and cancer registry incidence data (14). In addition, the CodCorrect algorithm adjusted the single cause estimates to fit into the separate all-cause mortality (15). The final mortality measures were used to estimate the incidence by the mortality-to-incidence ratio. YLDs were estimated by dividing the prevalence of breast cancer into four sequelae, including diagnosis and treatment, remission, metastasis, and terminal stages. Each sequela prevalence was then multiplied by a disability weight to estimate YLDs. YLLs were calculated by multiplying the number of deaths by age with the normative life expectancy at that age. DALYs were represented by the sum of YLDs and YLLs; one DALY can be interpreted as 1 year of “healthy life” lost.

The age-standardized rates, including age-standardized incident rates (ASIRs), age-standardized mortality rates (ASMRs), age-standardized DALY rates, and estimated annual percentage change (EAPC), were used to quantify the breast cancer burden trends (16). It is necessary to standardize the data when comparing several population groups with different age classes or for the same population over time in which the age profiles change. The EAPC was used to estimate the age-standardized rate trend over a specified interval. If the EAPC and the lower boundary of its 95% confidential interval (CI) were both greater than 0, the age-standardized rates were considered as increasing over the selected years. In addition, an unsupervised hierarchical cluster analysis was applied to classify the countries and territories according to their EAPC of ASIR, ASMR, and age-standardized DALY rates.

### Estimation of Attributable Burden

The GBD study proposed an attributable burden formula to quantify the burden of diseases and impairments attributable to 84 behavioral, environmental and occupational, and metabolic risk factors (17, 18). Four components were used to estimate the attributable burden of causes to risk factors, including the estimate of the burden metric being assessed (i.e., number of deaths, YLLs, YLDs, or DALYs), the levels of exposure for the risk factor, the counterfactual level of risk factor exposure, and the relative risk of the outcome caused by the exposure. The GBD comparative risk assessment summarized and updated 476 risk–outcome pairs supported by convincing or probable evidence in 2017, and we identified all five risk factors listed as paired outcomes of breast cancer, including alcohol use, high body mass index (BMI), high fasting plasma glucose, low physical activity, and tobacco smoking (19–25).

### Statistical Analysis

Two-sided *P* < 0.05 were considered statistically significant. Moreover, the uncertainty intervals (UIs) were calculated to quantify the effects of uncertainty on cause-specific estimation mortality, mainly brought by specifications of the cause-specific model, varied availability of data, and diversity of sample size. The 95% UIs were calculated with the 2.5th and 97.5th percentiles and all the estimates were reported next to each point estimate (9).

## Results

### Global Burden of Breast Cancer

There were 1,960,682 incident breast cancer cases (95% UI: 1,891,447–2,023,170) and 611,625 deaths (95% UI: 589,197–640,680) globally in 2017, contributing to 17,708,600 DALYs (95% UI: 16,899,498–18,674,972) (Table 1). Breast cancer was the second most common incident cancer and the fifth most common cause of cancer-related death worldwide. For women around the world, breast cancer was the most common cancer and the leading cause of cancer-related death (Supplementary Figure 1). The number of breast cancer cases increased by 123.14% (95% UI: 104.06–135.62%) from 1990 to 2017, and the EAPC of ASIR was 0.41 (95% CI: 0.35–0.47). Breast cancer led to a 74.96% (95% UI: 57.34–87.02%) increase in deaths in 2017 compared to 1990, with the ASMR decreasing from 8.66 (95% UI: 8.28–9.37) in 1990 to 7.65 (95% UI: 7.37–8.01) per 100,000 persons in 2017 (EAPC: −0.62; 95% CI: −0.68 to −0.55). DALYs increased by 69.73% (95% UI: 49.96–84.04%) during the study period, with a steeper increase for YLDs than for YLLs (124.42 vs. 66.49% increase) (Supplementary Table 2).

**Table 1:** Breast cancer incident cases, age-standardized incidence rate, deaths, age-standardized mortality rate, DALYs, and age-standardized DALY rates in 2017.

**Characteristics**	**Incident cases (95% UI)**	**ASIR per 10^**5**^ (95% UI)**	**Deaths (95% UI)**	**ASMR per 10^**5**^ (95% UI)**	**DALYs (95% UI)**	**Age-standardized DALY rates per 10^**5**^ (95% UI)**
**Overall**	1,960,682 (1,891,447–2,023,170)	24.19 (23.33–24.96)	611,625 (589,197–640,680)	7.65 (7.37–8.01)	17,708,600 (16,899,498–18,674,972)	216.29 (206.40–228.10)
**Sex**						
Female	1,937,574 (1,868,019–2,000,363)	45.91 (44.24–47.40)	600,728 (578,725–629,932)	14.15 (13.63–14.84)	17,423,143 (16,617,464–18,378,225)	414.67 (395.49–437.57)
Male	23,108 (22,258–23,999)	0.61 (0.59–0.54)	10,897 (10,467–11,370)	0.30 (0.29–0.31)	285,457 (272,793–299,429)	7.28 (6.96–7.64)
**SDI**						
High	781,346 (757,525–804,401)	40.99 (39.76–42.21)	181,004 (176,078–186,127)	8.42 (8.18–8.64)	4,290,889 (4,080,856–4,514,674)	231.16 (219.95–243.13)
High-middle	435,967 (400,352–458,737)	23.93 (22.00–25.15)	123,362 (114,318–128,240)	6.89 (6.38–7.16)	3,536,312 (3,259,624–3,720,805)	192.30 (177.03–202.40)
Middle	418,855 (378,165–444,111)	17.86 (16.12–18.92)	142,495 (128,467–150,361)	6.32 (5.70–6.67)	4,433,924 (3,996,316–4,723,874)	185.94 (167.24–197.93)
Low-middle	222,633 (199,262–275,621)	16.50 (14.75–20.62)	108,004 (95,987–136,991)	8.59 (7.62–10.99)	3,569,694 (3,178,209–4,459,868)	257.20 (229.12–322.56)
Low	94,422 (86,884–102,674)	11.62 (10.70–12.70)	54,826 (50,496–59,894)	7.31 (6.72–7.95)	1,819,824 (1,680,310–1,980,751)	214.72 (198.42–233.67)
**Region**						
Central Asia	16,634 (15,527–17,814)	19.66 (18.43–21.00)	6,164 (5,803–6,543)	7.89 (7.44–8.34)	198,308 (185,440–212,929)	229.30 (214.85–245.17)
East Asia	386,509 (326,128–417,015)	18.41 (15.49–19.86)	93,555 (78,460–100,095)	4.55 (3.81–4.87)	2,824,713 (2,409,605–3,047,356)	133.01 (112.47–143.55)
South Asia	210,811 (182,317–248,931)	14.07 (12.17–16.61)	108,966 (93,488–131,457)	7.79 (6.68–9.37)	3,487,738 (2,996,165–4,220,998)	226.32 (194.37–273.77)
Southeast Asia	125,590 (115,233–135,934)	18.70 (17.22–20.19)	50,881 (47,153–55,081)	8.08 (7.51–8.77)	1,706,010 (1,574,939–1,845,127)	248.40 (229.77–268.24)
High-income Asia Pacific	91,895 (85,833–97,839)	27.03 (25.31–28.75)	18,580 (17,800–19,405)	4.78 (4.57–5.00)	1,406,367 (1,332,094–1,489,128)	147.72 (138.55–156.79)
Australasia	18,761 (16,363–21,387)	44.22 (38.38–50.73)	4,131 (3,666–4,613)	8.91 (7.89–9.97)	104,618 (91,721–118,054)	252.16 (220.26–285.33)
Oceania	1,715 (1,274–2,379)	20.15 (15.82–26.69)	844 (650–1,148)	11.59 (9.48–14.66)	30,998 (22,602–44,128)	340.73 (260.95–466.45)
High-income North America	276,898 (266,909–287,822)	49.54 (47.62–51.52)	55,418 (53,745–57,196)	9.26 (8.97–9.57)	1,406,367 (1,332,094–1,489,128)	259.44 (245.38–274.95)
Caribbean	14,186 (12,637–15,935)	27.85 (24.80–31.29)	5,149 (4,528–5,853)	10.10 (8.87–11.49)	144,948 (124,816–168,503)	285.17 (245.66–332.08)
Andean Latin America	8,293 (7,251–9,587)	14.77 (12.92–17.07)	3,260 (2,881–3,732)	5.93 (5.24–6.78)	95,827 (83,870–110,884)	169.62 (148.52–196.28)
Central Latin America	51,422 (48,742–54,158)	21.09 (20.01–22.21)	15,682 (14,949–16,453)	6.60 (6.29–6.91)	473,911 (448,947–499,652)	191.52 (181.51–201.81)
Southern Latin America	22,659 (20,245–25,512)	28.78 (25.69–32.45)	9,041 (8,131–10,150)	11.10 (9.97–12.47)	219,614 (195,247–248,453)	281.38 (250.17–318.81)
Tropical Latin America	54,359 (52,537–56,239)	22.52 (21.79–23.28)	19,216 (18,734–19,702)	8.14 (7.94–8.34)	565,785 (548,115–583,686)	231.54 (224.47–238.82)
Central Europe	60,364 (57,627–63,445)	32.05 (30.60–33.70)	20,536 (19,735–21,373)	9.96 (9.57–10.37)	488,442 (466,009–513,027)	259.54 (247.43–272.86)
Eastern Europe	97,209 (93,529–100,843)	30.64 (29.43–31.85)	32,586 (31,689–33,542)	9.72 (9.45–10.01)	876,136 (845,015–910,073)	275.74 (265.49–286.83)
Western Europe	341,848 (324,867–358,885)	45.41 (43.21–47.65)	88,977 (84,917–93,164)	10.00 (9.55–10.47)	1,939,811 (1,818,466–2,056,232)	262.95 (246.66–279.56)
Central Sub-Saharan Africa	8,148 (6,236–10,695)	13.32 (10.70–17.01)	5,083 (3,970–6,576)	9.23 (7.59–11.51)	168,772 (126,775–222,327)	256.74 (199.98–332.62)
Eastern Sub-Saharan Africa	25,615 (22,320–29,467)	12.95 (11.36–14.82)	15,068 (13,192–17,331)	8.58 (7.55–9.77)	524,137 (457,901–605,323)	248.79 (217.84–286.48)
North Africa and Middle East	91,173 (85,206–100,561)	18.06 (16.90–20.28)	27,709 (25,885–31,151)	5.88 (5.52–6.72)	969,604 (900,819–1,070,338)	184.75 (172.30–204.52)
Southern Sub-Saharan Africa	10,482 (9,508–11,381)	17.44 (15.75–18.82)	5,469 (4,944–5,883)	9.91 (9.55–10.47)	160,614 (147,118–174,089)	256.68 (234.16–277.10)
Western Sub-Saharan Africa	46,110 (35,073–60,972)	20.57 (15.80–26.95)	25,311 (19,602–32,925)	12.50 (9.78–15.99)	848,382 (649,940–1,116,388)	362.75 (279.85–472.84)

*ASIRs, age-standardized incident rates; ASMR, age-standardized mortality rates; DALY, disability adjusted life-year; SDI, socio-demographic index; UI, uncertain interval*.

**Figure 1:**
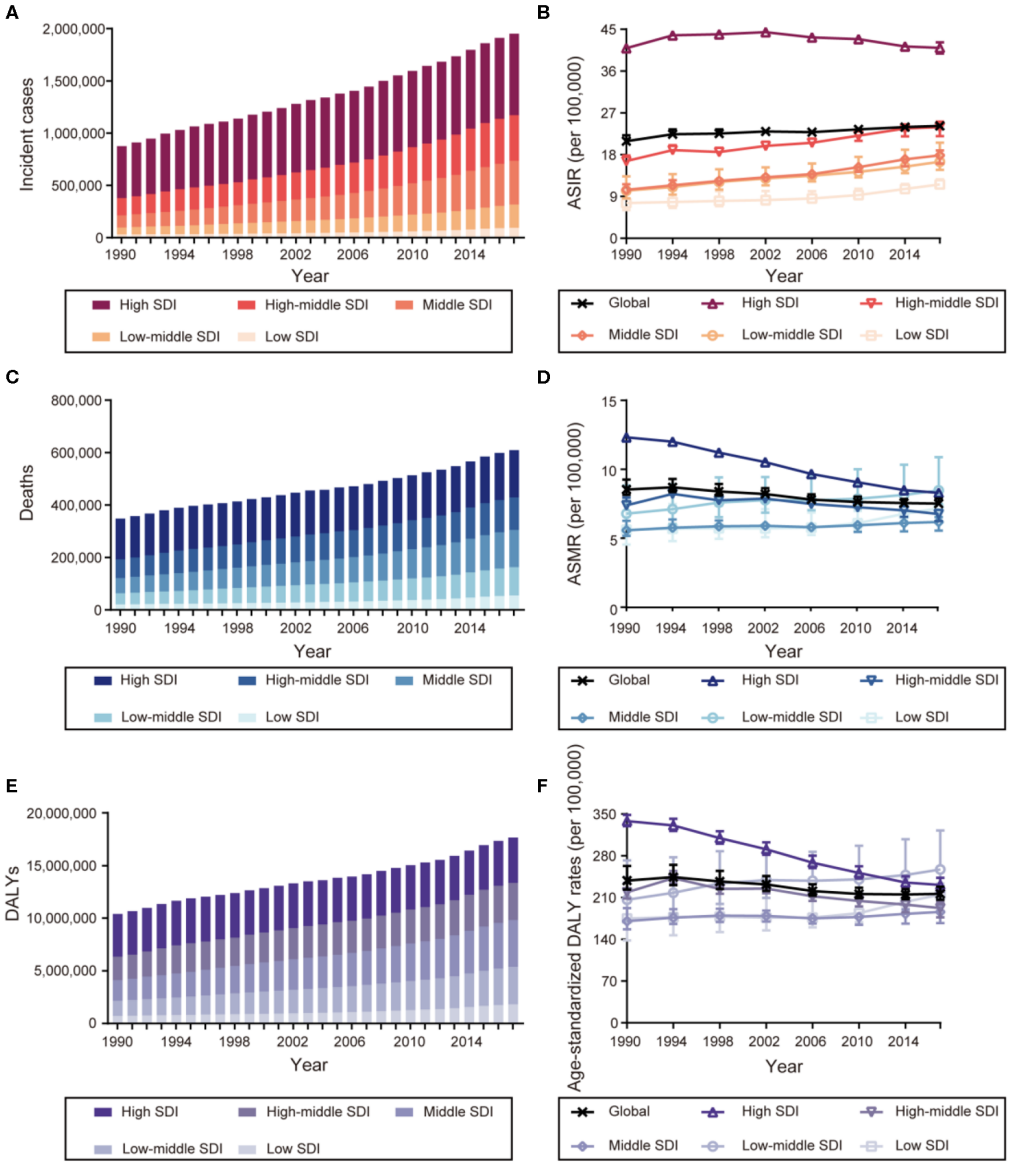
The burden and trends of breast cancer globally and in five sociodemographic index (SDI) quintiles from 1990 to 2017. **(A)** Incident cases. **(B)** Age-standardized incident rates (ASIRs). **(C)** Deaths. **(D)** Age-standardized mortality rates (ASMRs). **(E)** Disability-adjusted life-years (DALYs). **(F)** Age-standardized DALY rates.

Figure 1 shows that the incident cases of breast cancer increased in all SDI countries, with the highest increase in middle SDI countries (2.62-fold) and the lowest increase in high SDI countries (0.57-fold). The ASIR increased in countries in the high SDI regions until the early 2000s and began to fall thereafter, while they increased in other four SDI countries between 1990 and 2017. The number of deaths increased in all SDI countries, with the high SDI countries having the highest number of breast cancer deaths in 2017 [181,004 deaths (95% UI: 176,078–186,127)]. However, the ASMR decreased in the high and high-middle SDI countries. The DALYs increased in all SDI countries, though the rise was steeper in low and low-middle SDI countries (1.49- and 1.50-fold, respectively) and less prominent in the high SDI countries (0.06-fold). In addition to the ASMR, the age-standardized DALY rates of breast cancer also decreased in the high and high-middle SDI countries. Moreover, the proportions of the burden associated with YLLs and YLDs changed during the study period, with an increasing fraction of DALYs attributable to YLDs and a decreasing fraction to YLLs. The age-standardized DALY rates in high SDI countries due to YLDs increased from 8.48% in 1990 to 12.74% in 2017, while the trend was less prominent in low SDI countries (Supplementary Figure 2).

### Regional and National Burden of Breast Cancer

**Figure 2:**
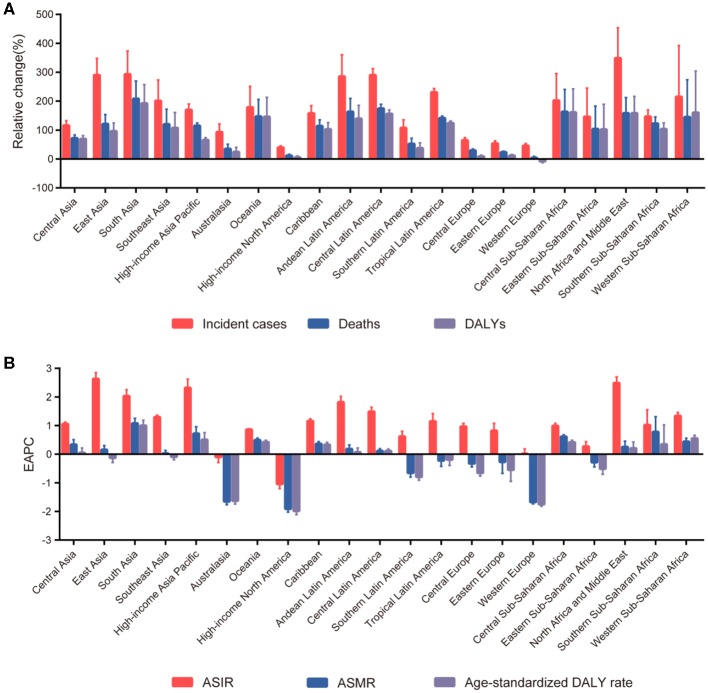
The trends in incidence, mortality, and disability-adjusted life-years (DALYs) of breast cancer at the regional level between 1990 and 2017. **(A)** The relative change in incident cases, deaths, and DALYs. **(B)** The estimated annual percentage changes (EAPCs) of age-standardized incident rate (ASIR), age-standardized mortality rate (ASMR), and age-standardized DALY rate.

**Figure 3:**
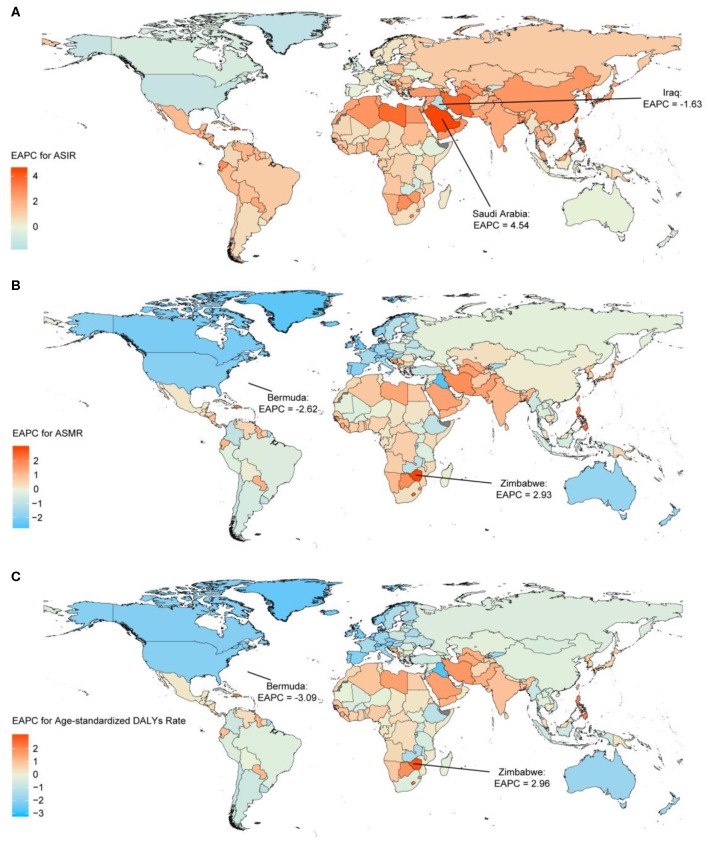
The estimated annual percentage changes (EAPCs) of breast cancer in 195 countries and territories between 1990 and 2017. **(A)** The EAPC of age-standardized incident rates (ASIRs). **(B)** The EAPC of age-standardized mortality rates (ASMRs). **(C)** The EAPC of age-standardized disability-adjusted life-year (DALY) rates. Countries with an extreme number of cases were annotated.

The highest absolute number of breast cancer incident cases was estimated in East Asia, with 386,508 cases (95% UI: 326,128–417,015) in 2017, while high-income North America contributed the highest ASIR of 49.54 per 100,000 (95% UI: 47.62–51.52) (Table 1). Breast cancer incident cases increased in all the geographical regions between 1990 and 2017, with the greatest increases observed in North Africa and Middle East (348.83%, 95% UI: 221.73–453.88%), followed by South Asia and Central Latin America. As for the ASIR, the most significant decrease was detected in high-income North America (EAPC: −1.04; 95% CI: −1.20 to −0.88). The most significant increase was detected in East Asia (EAPC: 2.63, 95% CI: 2.42–2.85) (Figure 2). Approximately 20% of the newly diagnosed cancer cases were recorded in China in 2017 (363,857, 95% UI: 304,358–394,316), followed by the USA and India (Supplementary Figure 3 and Supplementary Table 3). The largest increase in the ASIR was observed in Saudi Arabia (EAPC: 4.47; 95% CI: 4.47–4.61). There were 25 countries or territories that reported decreasing breast cancer ASIR between 1990 and 2017 (Figure 3A).

Most deaths occurred in South Asia in 2017 (108,966, 95% UI: 93,488–131,457), and the highest increase in breast cancer deaths was also in South Asia, with a 208.26% increase (95% UI: 120.90–269.39%). The highest ASMR was detected in Western Sub-Saharan Africa in 2017 (12.50, 95% UI: 9.78–15.99) (Table 1). The ASMR trends varied among the different regions during the study period. Among 21 geographical regions, eight regions, including high-income North America (EAPC: −1.91, 95% CI: −2.03 to −1.80), Western Europe (EAPC: −1.68, 95% CI: −1.74 to −1.61), and Australasia (EAPC: −1.65, 95% CI: −1.77 to −1.54), experienced decreases. The most significant increase was detected in South Asia (EAPC: 1.08, 95% CI: 0.90–1.25), followed by Southern Sub-Saharan Africa and high-income Asia Pacific (Figure 2). China, USA, and India, which had the largest populations worldwide, had the most deaths due to breast cancer in 2017 (Supplementary Figure 4 and Supplementary Table 3). The EAPC of ASMR between 1990 and 2017 was highest in Zimbabwe (EAPC: 2.93; 95% CI: 2.08–3.80) (Figure 3B).

South Asia, East Asia, and Western Europe were the areas with the highest DALYs due to breast cancer in 2017, while Western Sub-Saharan Africa and Oceania had the highest age-standardized DALY rates (Table 1). Except for Western Europe, the DALYs increased in all regions, ranging from a 5.85% increase (95% UI: 1.60–10.13%) in high-income North America to a 192.44% increase (95% UI: 107.68–257.46%) in South Asia. A notable decline was evident in the age-standardized DALY rates of high-income North America, with an EAPC of −1.99 (95% CI: −2.11 to −1.86) during the 27-year period (Figure 2). The highest total DALYs were observed in China, USA, and India, whereas the Bahamas, Nigeria, and Tonga had the highest age-standardized DALY rates in 2017 (Supplementary Figure 5 and Supplementary Table 3). Figure 3C shows that the highest EAPC of age-standardized DALY rates during the study period was observed in Zimbabwe (EAPC: 2.96; 95% CI: 2.06–3.87).

The unsupervised hierarchical cluster analysis classified the countries and territories into four clusters (Supplementary Figure 6). Twenty-five countries or territories, including South Korea, Iran, and Saudi Arabia, were classified in the “significant increase in all EAPCs” cluster. Forty-eight countries or territories, including Qatar, Brazil, and Poland, were classified in the “minor increase in EAPC of ASIR and minor decrease in EAPC of ASMR and age-standardized DALY rates” cluster. Eighty-three countries or territories, including Japan, Mexico, China, and India, were categorized into the “minor increase in all EAPCs” cluster. The remaining 39 countries or territories, including the UK, USA, France, and Germany, were categorized into the “significantly decrease in all EAPCs” cluster.

### Factors Associated With Estimated Annual Percentage Change

**Figure 4:**
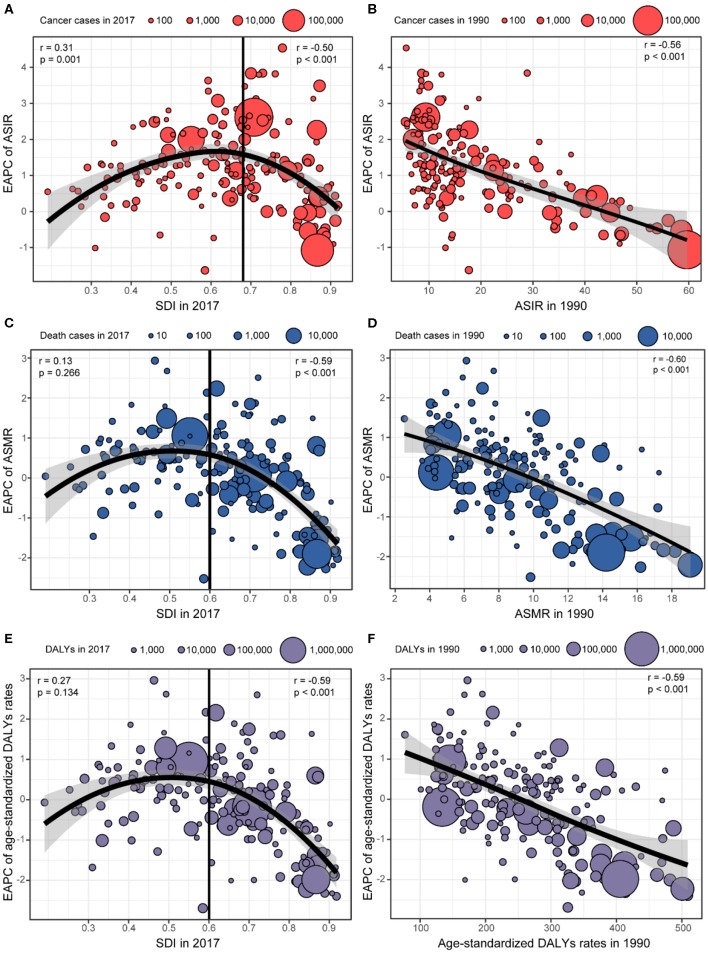
The association between estimated annual percentage changes (EAPCs) and age-standardized rates in 1990 and sociodemographic index (SDI) in 2017. **(A)** EAPC of age-standardized incident rates (ASIRs) and SDI in 2017. **(B)** EAPC of ASIR and ASIR in 1990. **(C)** EAPC of age-standardized mortality rates (ASMRs) and SDI in 2017. **(D)** EAPC of ASMR and ASMR in 1990. **(E)** EAPC of age-standardized disability-adjusted life-year (DALY) rates and SDI in 2017. **(F)** EAPC of age-standardized DALY rate and age-standardized DALY rates in 1990.

Figure 4 shows that there were significant associations between the EAPC and age-standardized rates in 1990, SDI in 2017, respectively. A significant positive association was detected between the EAPC of ASIR and the SDI (*r* = 0.31, *p* = 0.001) when the SDI was limited below 0.68. In contrast, for SDI above 0.68, countries with higher SDI experienced more rapid decreases in the ASIR of breast cancer from 1990 to 2017 (*r* = −0.50, *p* < 0.001; Figure 4A). A significant negative correlation was also observed between the EAPC of ASMR and the SDI (*r* = −0.59, *p* < 0.001) and the EAPC of age-standardized DALYs rates and the SDI (*r* = −0.59, *p* < 0.001) when the SDI was above 0.60. However, the correlation disappeared for SDI below 0.60 (Figures 4C,E). A significant negative association was found between the EAPC and age-standardized rates in 1990, regardless of ASIR, ASMR, or age-standardized DALY rates (Figures 4B,D,F).

### Attributable Burden of Breast Cancer to Risk Factors

**Figure 5:**
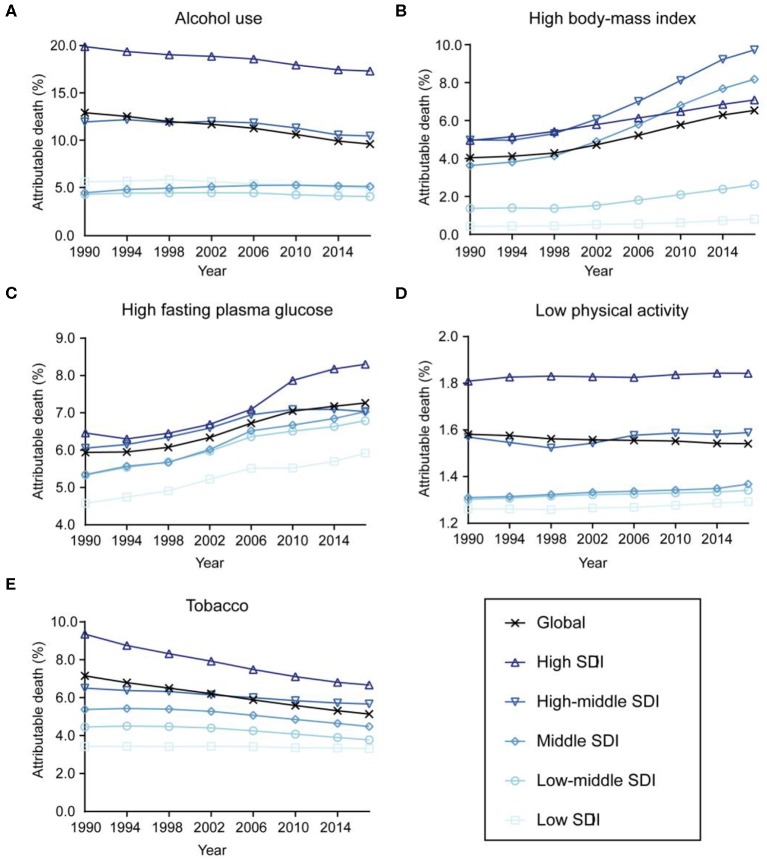
Contributions of different risk factors to breast cancer deaths globally and in five sociodemographic index (SDI) quintiles from 1990 to 2017. **(A)** Alcohol use. **(B)** High body mass index. **(C)** High fasting plasma glucose. **(D)** Low physical activity. **(E)** Tobacco.

The contributions of different risk factors to breast cancer deaths are demonstrated in Figure 5. Alcohol use accounted for 9.64% (95% UI: 7.99–11.26%) of the global deaths in 2017 and showed a decreasing trend in most countries between 1990 and 2017, except for middle SDI countries. Breast cancer deaths attributable to high fasting plasma glucose and high BMI increased globally and in all SDI countries during the study period. Deaths of breast cancer attributable to low physical activity remained stable, while the percentages of tobacco-attributable breast cancer deaths decreased from 1990 to 2017.

## Discussion

This study presents the recent burden and trends of incidence, mortality, and DALYs associated with breast cancer at the global, regional, and national levels based on the GBD 2017 results. Our analysis revealed that breast cancer remained a major public health concern globally, and the trends of incidence, mortality, and DALYs were heterogeneous across regions and countries. Age-standardized rates in 1990 and SDI in 2017 were both associated with EAPC. Moreover, the changes of the attributable burden to different breast cancer risk factors between 1990 and 2017 also varied.

In general, our analysis found that both the breast cancer incidence and number of cases increased from 1990 to 2017. However, the temporal trends of breast cancer incidence varied by region and country mainly based on socioeconomic status. For example, in high SDI countries, such as the USA, Canada, and Australia, the ASIR began to fall after 2000 partly owing to declines in the use of postmenopausal hormonal treatments after the findings linked postmenopausal hormone use to increased breast cancer risk, as first reported in the USA (26, 27). Nevertheless, in lower SDI countries, the ASIR increased throughout the study period. The widespread use of breast mammography screening has been associated with the increased incidence of breast cancer around the world (28, 29). A high prevalence of mammography screening in higher SDI countries may cause increased ASIR in these countries. The increased incidence of breast cancer in lower SDI countries may have resulted from lifestyle westernization, characterized by a change in reproductive patterns including a relatively late first birth, a young age at menarche, a short duration of breastfeeding, low parity, and obesity (30, 31). Moreover, with widespread screening and an increased life expectancy in lower SDI countries, the incidence of breast cancer is expected to increase in future years.

Consistent with the increase in incident cases, we also noted an increase in breast cancer deaths at the global level. The ASMR of breast cancer have been significantly decreasing in high and high-middle SDI regions, such as Western Europe, high-income North America, and Australasia. The decline in ASMR might be attributable to recent improvements in therapeutic approaches, including surgical technology, radiation therapy, and systemic treatment. An increasing number of novel drugs, including cyclin-dependent kinase 4/6 inhibitors, poly ADP-ribose polymerase inhibitors, and programmed death-ligand 1 inhibitors, have been shown to improve outcomes and have been recently approved for breast cancer treatment in the past few years (32). However, the mortality rate still revealed a relatively constant or slightly increasing trend in low, low-middle, and middle SDI countries over the last several decades. This variation among countries may result from differences in accessibility to novel drugs and facilities, as well as the application of clinical guidelines (33).

Interestingly, our study revealed that in higher SDI countries, the age-standardized DALY rates have decreased but the proportions of the burden associated with YLDs have increased. The increase in YLDs in high-income countries is consistent with the overdiagnosis and overtreatment of some breast cancers (34). Moreover, we found that the age-standardized DALY rates increased in lower SDI countries with a stable proportion of DALYs attributable to YLLs and YLDs. The underdiagnosis of aggressive breast cancers may have caused this phenomenon.

Furthermore, we found that the EAPC of ASIR, ASMR, or age-standardized DALY rates between 1990 and 2017 was significantly negatively associated with the baseline rates in 1990. This result might be partly explained by the fact that increased baseline rates are correlated with low amounts of significant rate variations. The other reason is that countries with low age-standardized rates are more likely to consider breast cancer as a low priority in disease prevention and treatment programs compared with other public health issues.

Alcohol use is an important risk factor contributing to the incidence and mortality of many kinds of cancer, including breast cancer (20). A strong and consistent dose-response relationship between alcohol and breast cancer has been revealed, even at low levels of consumption (19). Since 1990, alcohol use has decreased in most high-income countries, with the largest reductions recently achieved in Eastern Europe. The contribution of high-income countries to global alcohol use decreased by 16% from 1990 to 2017 (35). This finding is consistent with the observed decreases in the incidence trends of breast cancer in high SDI countries, as well as the decreasing contribution of alcohol use to the estimated number of breast cancer deaths in these countries. Another risk factor is tobacco use, and the relative risk was found to be 1.13, 1.10, and 1.07 for current active smoking, ever active smoking, and ever passive smoking, respectively (25). It has been observed that the global prevalence of daily tobacco smoking has declined significantly, which also matches the results of our analysis (36). Moreover, our study showed a rapidly increasing contribution of high BMI and high fasting plasma glucose globally between 1990 and 2017, indicating that it is necessary for policymakers to promote the maintenance of healthy body weight and serum glucose level to reduce the risk of breast cancer (37).

Our results are in line with previous publications that analyzed other data sources (38–41). For example, the latest World Cancer Report in 2020, which includes 2.1 million new cases in 2018, also revealed that the breast cancer incidence increased during the past several years (41). However, the GBD study provides all estimated data from 1990 to 2017 and employs a specific composite measure of disease burden in terms of DALYs. Furthermore, the GBD study allows a comprehensive analysis of breast cancer epidemiology in all countries, while prior studies which used the other datasets have focused on one aspect of epidemiology, such as incidence or mortality trends, in selected countries. Although we also found that several similar researches have measured the disease burden of breast cancer based on the GBD study, there are some differences between our analysis and these researches (42–45). We revealed that there were significant associations between the EAPC and age-standardized rates in 1990, SDI in 2017, respectively. ASIR, ASMR, and age-standardized DALY rates and their corresponding EAPCs were all included in our analysis, while past researches have only focused on the correlation of EAPC and ASIR in 1990 (42, 43). Our analysis also demonstrated the contributions of different risk factors to breast cancer deaths, including alcohol use, high BMI, high fasting plasma glucose, low physical activity, and tobacco use. Only one research included a similar analysis, but it missed two risk factors associated with breast cancer deaths: low physical activity and tobacco use (42). In addition, we applied the unsupervised hierarchical cluster analysis to classify the countries and territories into four clusters. This analysis revealed the different breast cancer burden and trends in different nations and the allocation of appropriate health care resources for breast cancer prevention, screening, and treatment could be considered according to this analysis. Moreover, our analysis revealed that the proportions of the burden associated with YLLs and YLDs changed during the study period, with an increasing fraction of DALYs attributable to YLDs and a decreasing fraction to YLLs.

Our analysis estimated the disease burden and trends of breast cancer at the global, regional, and national levels in many aspects, including the number of incident cases, deaths, DALYs, age-standardized rates, EAPCs, factors associated with EAPCs, and attributable burden to breast cancer risk factors. However, several limitations should also be noted when interpreting our results. First, although several modeling methods were applied to analyze the GBD data, the low quality of data sources in several countries may have affected the produced estimates. For example, underreporting breast cancer on death certificates and underestimating breast cancer due to a lack of medical information may cause some fluctuations in the incidence and mortality rates. Second, breast cancer is now classified into several molecular subtypes according to the expression of hormone receptors and HER2 (46). Their epidemiological features, as well as risk factors, recurrence patterns, and outcomes, are not the same (47–49). However, due to the lack of relevant pathological data, the burden and trends of breast cancer stratified by molecular subtype or histology were not assessed in the current study.

In conclusion, breast cancer remains a major public health burden worldwide. In the past 30 years, the incident cases of breast cancer, as well as death cases and DALYs, have increased globally. As prevalent cases are early breast cancer identified during early screening rounds and routine breast screening is becoming more popular in less developed regions, the incidence is expected to increase temporarily in the near term. On the other hand, the breast cancer burden trends vary among countries based on socioeconomic status. Breast cancer mortality and DALY rates are decreasing in developed countries while still increasing in developing countries mainly due to differences in accessibility to novel drugs and the application of clinical guidelines. The information provided in this study should help to illustrate the global disease burden and trends of breast cancer and guide the allocation of appropriate health care resources for breast cancer prevention, screening, and treatment.

## Data Availability Statement

Publicly available datasets were analyzed in this study. This data can be found here: http://ghdx.healthdata.org/gbd-results-tool.

## Author Contributions

PJ, YG, G-HD, and Z-MS designed the project. PJ and YG equally contributed to perform the data analyses and to write the manuscript. M-LJ acquired the original data and developed the methodology. XH contributed to conceptualizing the study and edited the manuscript. G-HD and Z-MS equally supervised the overall work. All authors read and approved the final manuscript.

## Conflict of Interest

The authors declare that the research was conducted in the absence of any commercial or financial relationships that could be construed as a potential conflict of interest.
