# Supplementary material for: The Burden and Trends of Breast Cancer From 1990 to 2017 at the Global, Regional, and National Levels: Results From the Global Burden of Disease Study 2017
Source: Front Oncol. 2020 May 12;10:650. doi: 10.3389/fonc.2020.00650 (PMC7247846; doi:10.3389/fonc.2020.00650)
Supplement: Supplementary file 1 [file Data_Sheet_1.PDF]

Supplementary Material

Supplementary Table 1: Socio-demographic index (SDI) groupings by geography, based on 2017 values

Supplementary Table 2: The trends in incidence, mortality and DALYs of breast cancer globally and in 5 SDI quintiles between 1990 and 2017

Supplementary Table 3: Breast cancer incident cases, age-standardized incidence rate, deaths, age-standardized mortality rate, DALYs, and age-standardized DALY rates in 2017 at the national level

Supplementary Figure 1: Breast cancer ranking by total incidence, mortality and DALYs at global, regional, and national levels in 2017

Supplementary Figure 2: Age-standardized DALY rates from breast cancer YLDs and YLLs globally and in 5 SDI quintiles

Supplementary Figure 3: The global disease burden of breast cancer incidence in 195 countries and territories

Supplementary Figure 4: The global disease burden of breast cancer mortality in 195 countries and territories

Supplementary Figure 5: The global disease burden of breast cancer DALYs in 195 countries and territories

Supplementary Figure 6: The clusters of countries and territories according to the EAPC of breast cancer ASIR, ASMR and age-standardized DALY rates between 1990 and 2017

Supplementary Table 1: Socio-demographic index (SDI) groupings by geography, based on 2017 values

Location Name	SDI Quintile
Aichi	High SDI
Akita	High SDI
Alabama	High SDI
Alaska	High SDI
Andorra	High SDI
Aomori	High SDI
Arizona	High SDI
Arkansas	High SDI
Australia	High SDI
Austria	High SDI
Belgium	High SDI
Brunei	High SDI
California	High SDI
Canada	High SDI
Chiba	High SDI
Colorado	High SDI
Connecticut	High SDI
Croatia	High SDI
Cyprus	High SDI
Czech Republic	High SDI
Delaware	High SDI
Denmark	High SDI
District of Columbia	High SDI
East Midlands	High SDI
East of England	High SDI
Ehime	High SDI
England	High SDI
Estonia	High SDI
Finland	High SDI
Florida	High SDI
France	High SDI
Fukui	High SDI
Fukuoka	High SDI
Fukushima	High SDI
Georgia	High SDI
Germany	High SDI
Gifu	High SDI
Greater London	High SDI
Greece	High SDI
Gunma	High SDI
Hawaii	High SDI

Hiroshima	High SDI
Hokkaido	High SDI
Hyogo	High SDI
Ibaraki	High SDI
Iceland	High SDI
Idaho	High SDI
Illinois	High SDI
Indiana	High SDI
Iowa	High SDI
Ireland	High SDI
Ishikawa	High SDI
Italy	High SDI
Iwate	High SDI
Japan	High SDI
Kagawa	High SDI
Kagoshima	High SDI
Kanagawa	High SDI
Kansas	High SDI
Kentucky	High SDI
Kochi	High SDI
Kumamoto	High SDI
Kyoto	High SDI
Latvia	High SDI
Lithuania	High SDI
Louisiana	High SDI
Luxembourg	High SDI
Maine	High SDI
Malta	High SDI
Maryland	High SDI
Massachusetts	High SDI
Michigan	High SDI
Mie	High SDI
Minnesota	High SDI
Mississippi	High SDI
Missouri	High SDI
Miyagi	High SDI
Miyazaki	High SDI
Montana	High SDI
Nagano	High SDI
Nagasaki	High SDI
Nara	High SDI
Nebraska	High SDI
Netherlands	High SDI
Nevada	High SDI

New Hampshire	High SDI
New Jersey	High SDI
New Mexico	High SDI
New York	High SDI
New Zealand	High SDI
Niigata	High SDI
North Carolina	High SDI
North Dakota	High SDI
North East England	High SDI
North West England	High SDI
Northern Ireland	High SDI
Norway	High SDI
Ohio	High SDI
Oita	High SDI
Okayama	High SDI
Okinawa	High SDI
Oklahoma	High SDI
Oregon	High SDI
Osaka	High SDI
Pennsylvania	High SDI
Poland	High SDI
Rhode Island	High SDI
Saga	High SDI
Saitama	High SDI
Scotland	High SDI
Shiga	High SDI
Shimane	High SDI
Shizuoka	High SDI
Singapore	High SDI
Slovakia	High SDI
Slovenia	High SDI
South Carolina	High SDI
South Dakota	High SDI
South East England	High SDI
South Korea	High SDI
South West England	High SDI
Spain	High SDI
Stockholm	High SDI
Sweden	High SDI
Sweden except Stockholm	High SDI
Switzerland	High SDI
Taiwan	High SDI
Tennessee	High SDI
Texas	High SDI

Tochigi	High SDI
Tokushima	High SDI
Tokyo	High SDI
Tottori	High SDI
Toyama	High SDI
United Kingdom	High SDI
United States	High SDI
Utah	High SDI
Vermont	High SDI
Virginia	High SDI
Wakayama	High SDI
Wales	High SDI
Washington	High SDI
West Midlands	High SDI
West Virginia	High SDI
Wisconsin	High SDI
Wyoming	High SDI
Yamagata	High SDI
Yamaguchi	High SDI
Yamanashi	High SDI
Yorkshire and the Humber	High SDI
American Samoa	High-middle SDI
Antigua and Barbuda	High-middle SDI
Argentina	High-middle SDI
Armenia	High-middle SDI
Azerbaijan	High-middle SDI
Bahrain	High-middle SDI
Barbados	High-middle SDI
Belarus	High-middle SDI
Bermuda	High-middle SDI
Bosnia and Herzegovina	High-middle SDI
Bulgaria	High-middle SDI
Chile	High-middle SDI
China	High-middle SDI
Distrito Federal	High-middle SDI
East Kalimantan	High-middle SDI
Georgia	High-middle SDI
Greenland	High-middle SDI
Guam	High-middle SDI
Hungary	High-middle SDI
Iran	High-middle SDI
Israel	High-middle SDI
Jakarta	High-middle SDI
Kazakhstan	High-middle SDI

Kuwait	High-middle SDI
Lebanon	High-middle SDI
Libya	High-middle SDI
Macedonia	High-middle SDI
Malaysia	High-middle SDI
Mauritius	High-middle SDI
Montenegro	High-middle SDI
North Kalimantan	High-middle SDI
Northern Mariana Islands	High-middle SDI
Oman	High-middle SDI
Portugal	High-middle SDI
Puerto Rico	High-middle SDI
Qatar	High-middle SDI
Riau	High-middle SDI
Riau Islands	High-middle SDI
Rio de Janeiro	High-middle SDI
Romania	High-middle SDI
Russian Federation	High-middle SDI
Santa Catarina	High-middle SDI
Sao Paulo	High-middle SDI
Saudi Arabia	High-middle SDI
Serbia	High-middle SDI
The Bahamas	High-middle SDI
Turkey	High-middle SDI
Ukraine	High-middle SDI
United Arab Emirates	High-middle SDI
Uruguay	High-middle SDI
Virgin Islands, U.S.	High-middle SDI
Aceh	Middle SDI
Aguascalientes	Middle SDI
Albania	Middle SDI
Algeria	Middle SDI
Amapa	Middle SDI
Amazonas	Middle SDI
Baja California	Middle SDI
Baja California Sur	Middle SDI
Bali	Middle SDI
Bangka-Belitung Islands	Middle SDI
Banten	Middle SDI
Botswana	Middle SDI
Brazil	Middle SDI
Campeche	Middle SDI
Central Java	Middle SDI
Central Kalimantan	Middle SDI

Central Sulawesi	Middle SDI
Chiapas	Middle SDI
Chihuahua	Middle SDI
Coahuila	Middle SDI
Colima	Middle SDI
Colombia	Middle SDI
Costa Rica	Middle SDI
Cuba	Middle SDI
Dominica	Middle SDI
Durango	Middle SDI
East Java	Middle SDI
Ecuador	Middle SDI
Equatorial Guinea	Middle SDI
Espirito Santo	Middle SDI
Fiji	Middle SDI
Gabon	Middle SDI
Goias	Middle SDI
Grenada	Middle SDI
Guanajuato	Middle SDI
Guerrero	Middle SDI
Hidalgo	Middle SDI
Indonesia	Middle SDI
Jalisco	Middle SDI
Jamaica	Middle SDI
Jambi	Middle SDI
Jordan	Middle SDI
Lampung	Middle SDI
Maldives	Middle SDI
Mato Grosso	Middle SDI
Mato Grosso do Sul	Middle SDI
Mexico	Middle SDI
Mexico	Middle SDI
Mexico City	Middle SDI
Michoacan de Ocampo	Middle SDI
Minas Gerais	Middle SDI
Moldova	Middle SDI
Mongolia	Middle SDI
Morelos	Middle SDI
Namibia	Middle SDI
Nayarit	Middle SDI
North Sulawesi	Middle SDI
North Sumatra	Middle SDI
Nuevo Leon	Middle SDI
Oaxaca	Middle SDI

Panama	Middle SDI
Paraguay	Middle SDI
Parana	Middle SDI
Peru	Middle SDI
Philippines	Middle SDI
Puebla	Middle SDI
Queretaro	Middle SDI
Quintana Roo	Middle SDI
Rio Grande do Sul	Middle SDI
Rondonia	Middle SDI
Roraima	Middle SDI
Saint Lucia	Middle SDI
Saint Vincent and the Grenadines	Middle SDI
San Luis Potosi	Middle SDI
Sergipe	Middle SDI
Seychelles	Middle SDI
Sinaloa	Middle SDI
Sonora	Middle SDI
South Africa	Middle SDI
South Kalimantan	Middle SDI
South Sulawesi	Middle SDI
South Sumatra	Middle SDI
Sri Lanka	Middle SDI
Suriname	Middle SDI
Syria	Middle SDI
Tabasco	Middle SDI
Tamaulipas	Middle SDI
Thailand	Middle SDI
Tlaxcala	Middle SDI
Tocantins	Middle SDI
Tonga	Middle SDI
Trinidad and Tobago	Middle SDI
Tunisia	Middle SDI
Turkmenistan	Middle SDI
Uzbekistan	Middle SDI
Venezuela	Middle SDI
Veracruz de Ignacio de la Llave	Middle SDI
Vietnam	Middle SDI
West Java	Middle SDI
West Papua	Middle SDI
West Sumatra	Middle SDI
Yogyakarta	Middle SDI
Yucatan	Middle SDI
Zacatecas	Middle SDI

Acre	Low-middle SDI
Alagoas	Low-middle SDI
Angola	Low-middle SDI
Bahia	Low-middle SDI
Belize	Low-middle SDI
Bengkulu	Low-middle SDI
Bhutan	Low-middle SDI
Bolivia	Low-middle SDI
Cambodia	Low-middle SDI
Cameroon	Low-middle SDI
Cape Verde	Low-middle SDI
Ceara	Low-middle SDI
Congo	Low-middle SDI
Djibouti	Low-middle SDI
Dominican Republic	Low-middle SDI
East Nusa Tenggara	Low-middle SDI
Egypt	Low-middle SDI
El Salvador	Low-middle SDI
Federated States of Micronesia	Low-middle SDI
Ghana	Low-middle SDI
Gorontalo	Low-middle SDI
Guatemala	Low-middle SDI
Guyana	Low-middle SDI
Honduras	Low-middle SDI
India	Low-middle SDI
Iraq	Low-middle SDI
Kenya	Low-middle SDI
Kyrgyzstan	Low-middle SDI
Laos	Low-middle SDI
Lesotho	Low-middle SDI
Maluku	Low-middle SDI
Maranhao	Low-middle SDI
Marshall Islands	Low-middle SDI
Mauritania	Low-middle SDI
Morocco	Low-middle SDI
Myanmar	Low-middle SDI
Nicaragua	Low-middle SDI
Nigeria	Low-middle SDI
North Korea	Low-middle SDI
North Maluku	Low-middle SDI
Pakistan	Low-middle SDI
Palestine	Low-middle SDI
Papua	Low-middle SDI
Para	Low-middle SDI

Paraiba	Low-middle SDI
Pernambuco	Low-middle SDI
Piaui	Low-middle SDI
Rio Grande do Norte	Low-middle SDI
Samoa	Low-middle SDI
Sao Tome and Principe	Low-middle SDI
Southeast Sulawesi	Low-middle SDI
Sudan	Low-middle SDI
Swaziland	Low-middle SDI
Tajikistan	Low-middle SDI
Timor-Leste	Low-middle SDI
Vanuatu	Low-middle SDI
West Kalimantan	Low-middle SDI
West Nusa Tenggara	Low-middle SDI
West Sulawesi	Low-middle SDI
Zambia	Low-middle SDI
Zimbabwe	Low-middle SDI
Afghanistan	Low SDI
Bangladesh	Low SDI
Benin	Low SDI
Burkina Faso	Low SDI
Burundi	Low SDI
Central African Republic	Low SDI
Chad	Low SDI
Comoros	Low SDI
Cote d'Ivoire	Low SDI
Democratic Republic of the Congo	Low SDI
Eritrea	Low SDI
Ethiopia	Low SDI
Guinea	Low SDI
Guinea-Bissau	Low SDI
Haiti	Low SDI
Kiribati	Low SDI
Liberia	Low SDI
Madagascar	Low SDI
Malawi	Low SDI
Mali	Low SDI
Mozambique	Low SDI
Nepal	Low SDI
Niger	Low SDI
Papua New Guinea	Low SDI
Rwanda	Low SDI
Senegal	Low SDI
Sierra Leone	Low SDI

Solomon Islands	Low SDI
Somalia	Low SDI
South Sudan	Low SDI
Tanzania	Low SDI
The Gambia	Low SDI
Togo	Low SDI
Uganda	Low SDI
Yemen	Low SDI

Supplementary Table 2: The trends in incidence, mortality and DALYs of breast cancer globally and in 5 SDI quintiles between 1990 and 2017

Characteristics	Relative change in incident cases (95% UI)	EAPC of ASIR (95% CI)	Relative change in deaths (95% UI)	EAPC of ASMR (95% CI)	Relative change in DALYs (95% UI)	EAPC of age-standardized DALY rates (95% CI)
**Overall**	123.14% (104.06% to 135.62%)	0.41 (0.35 to 0.47)	74.96% (57.34% to 87.02%)	-0.62 (-0.68 to -0.55)	69.73% (49.96% to 84.04%)	-0.56 (-0.63 to -0.48)
**Sex**						
Female	122.66% (103.41% to 135.28%)	0.45 (0.38 to 0.51)	74.17% (56.44% to 86.37%)	-0.59 (-0.66 to -0.52)	69.05% (49.15% to 83.61%)	-0.58 (-0.66 to -0.49)
Male	171.75% (152.54% to 190.41%)	1.17 (1.01 to 1.34)	133.06% (112.79% to 153.64%)	0.57 (0.42 to 0.71)	124.32% (103.56% to 146.87%)	0.72 (0.55 to 0.89)
**SDI**						
High	57.08% (52.06% to 61.79%)	-0.13 (-0.25 to -0.01)	16.48% (13.05% to 19.68%)	-1.59 (-1.64 to -1.54)	5.63% (2.03% to 8.81%)	-1.58 (-1.64 to -1.53)
High-middle	163.11% (118.73% to 184.52%)	1.27 (1.17 to 1.37)	72.76% (46.19% to 84.08%)	-0.51 (-0.67 to -0.35)	59.30% (34.20% to 71.26%)	-0.73 (-0.88 to -0.58)
Middle	262.44% (188.11% to 305.56%)	1.95 (1.87 to 2.03)	144.64% (96.48% to 172.78%)	0.27 (0.20 to 0.33)	126.21% (82.70% to 154.77%)	0.17 (0.09 to 0.25)
Low-middle	228.33% (141.57% to 310.50%)	1.65 (1.58 to 1.72)	154.40% (85.42% to 218.23%)	0.64 (0.54 to 0.73)	149.61% (80.17% to 217.70%)	0.63 (0.52 to 0.74)
Low	214.59% (124.08% to 299.62%)	1.44 (1.22 to 1.67)	159.35% (85.72% to 223.84%)	0.75 (0.55 to 0.95)	149.04% (77.91% to 217.13%)	0.58 (0.39 to 0.78)

Abbreviations: ASIR, age-standardized incident rates. ASMR, age-standardized mortality rates. CI, confidential interval. DALY, disability adjusted life-year. EAPC, estimated annual percentage change. SDI, socio-demographic index. UI, uncertain interval.

Supplementary Table 3: Breast cancer incident cases, age-standardized incidence rate, deaths, age-standardized mortality rate, DALYs, and age-standardized DALY rates in 2017 at the national level

Countries	Incident cases, (95% UI)	ASIR per 105, (95% UI)	Deaths, (95% UI)	ASMR per 105, (95% UI)	DALYs, (95% UI)	Age-Standardized DALY rates per 105, (95% UI)
Afghanistan	2,225 (934-5,096)	14.32 (6.84-31.99)	1,296 (573-2,961)	9.15 (4.64-19.93)	49,822 (20,233-118,214)	300.97 (136.47-690.35)
Albania	706 (506-952)	19.45 (13.75-26.28)	224 (165-297)	5.74 (4.26-7.54)	6,710 (4,815-8,834)	182.27 (129.43-239.2)
Algeria	6,760 (5,793-7,722)	17.76 (15.3-20.15)	2,233 (1,939-2,497)	6.2 (5.41-6.92)	76,856 (65,547-87,084)	197.22 (170.07-222.55)
American Samoa	17 (14-19)	34.97 (29.95-40.3)	6 (5-7)	14.39 (12.5-16.22)	198 (170-229)	400.83 (342.91-457.19)
Andorra	61 (43-83)	46.47 (32.83-63.05)	14 (10-18)	9.77 (6.96-13.18)	343 (245-466)	263.91 (188.01-358.46)
Angola	1,792 (1,314-2,460)	13.29 (9.94-17.76)	1,049 (784-1,421)	8.76 (6.64-11.61)	36,141 (26,352-49,603)	250.54 (187.37-339.02)
Antigua and Barbuda	38 (34-42)	35.71 (31.93-39.76)	13 (12-14)	12.42 (11.38-13.55)	354 (318-393)	330.47 (298.1-365.82)
Argentina	15,729 (13,401-18,386)	30.72 (26.12-35.96)	6,602 (5,724-7,607)	12.48 (10.81-14.38)	159,858 (136,602-186,702)	315.84 (269.41-369.61)
Armenia	1,344 (1,228-1,467)	33.84 (30.92-36.97)	516 (480-550)	12.61 (11.75-13.48)	13,997 (12,984-15,071)	349.52 (325.95-376)
Australia	15,722 (13,394-18,442)	43.87 (37.14-51.79)	3,394 (2,922-3,869)	8.65 (7.42-9.89)	85,190 (72,847-98,406)	242.9 (206.81-281.58)
Austria	5,936 (5,351-6,592)	38.89 (34.87-43.3)	1,723 (1,577-1,877)	9.62 (8.8-10.51)	36,026 (32,696-39,585)	237.47 (214.81-261.87)
Azerbaijan	2,072 (1,737-2,451)	18.67 (15.74-21.78)	705 (604-815)	6.81 (5.88-7.84)	24,592 (20,645-28,829)	216.78 (184.15-252.11)
Bahrain	247 (206-296)	19.54 (16.24-23.07)	67 (57-79)	6.45 (5.43-7.54)	2,339 (1,989-2,777)	163.33 (139.41-189.72)
Bangladesh	13,981 (11,365-17,020)	10.31 (8.43-12.48)	6,927 (5,706-8,367)	5.4 (4.46-6.48)	222,893 (182,613-271,932)	161.07 (132.29-195.7)
Barbados	210 (183-236)	47.3 (41.12-53.3)	74 (65-82)	15.82 (13.99-17.62)	1,875 (1,644-2,107)	431.55 (378.28-484.66)
Belarus	3,511 (3,102-3,981)	23.91 (21.1-27.15)	1,145 (1,033-1,267)	7.36 (6.67-8.15)	30,723 (27,582-34,375)	208.38 (187.24-231.87)
Belgium	9,202 (8,232-10,282)	47.54 (42.46-53.28)	2,490 (2,260-2,724)	11.01 (9.95-12.03)	53,692 (48,402-59,239)	282.29 (254.02-311.67)

Belize	38 (33-42)	12.45 (11-13.94)	16 (14-17)	5.51 (4.93-6.09)	504 (446-565)	160.56 (142.59-179.34)
Benin	672 (489-873)	12.01 (8.84-15.47)	417 (306-537)	8.18 (6.15-10.46)	13,741 (10,009-17,856)	231.96 (170.15-299.51)
Bermuda	38 (33-44)	32.69 (27.93-37.85)	11 (10-13)	9.08 (7.89-10.34)	241 (206-276)	212.11 (181.46-243.99)
Bhutan	78 (51-140)	10.76 (7.11-19.06)	38 (26-69)	5.86 (3.98-10.59)	1,232 (813-2,224)	165.47 (111.59-297.25)
Bolivia	1,465 (1,069-1,932)	15.85 (11.55-20.91)	700 (520-913)	7.93 (5.94-10.3)	21,068 (15,287-28,043)	222.73 (162.34-294.04)
Bosnia and Herzegovina	1,452 (1,077-1,681)	26.62 (19.63-30.66)	574 (421-653)	9.92 (7.3-11.23)	14,666 (10,718-16,690)	266.03 (193.91-303.19)
Botswana	301 (243-366)	18.87 (15.44-22.86)	144 (119-174)	10.4 (8.6-12.5)	4,381 (3,551-5,310)	261.29 (213.95-316.5)
Brazil	53,174 (51,372-55,067)	22.56 (21.8-23.34)	18,725 (18,256-19,194)	8.12 (7.92-8.32)	551,306 (534,888-569,189)	231.13 (224.34-238.58)
Brunei	161 (131-189)	37.15 (30.65-42.86)	38 (32-43)	9.72 (8.3-10.95)	1,433 (1,196-1,663)	323.88 (272.8-371.68)
Bulgaria	4,351 (3,910-4,865)	35.07 (31.49-39.11)	1,380 (1,258-1,507)	10.14 (9.23-11.04)	35,007 (31,785-38,416)	290.38 (262.74-319)
Burkina Faso	1,853 (1,425-2,463)	16.68 (12.91-22.96)	1,079 (831-1,491)	10.83 (8.42-15.25)	38,274 (29,489-50,568)	325.71 (251.18-446.38)
Burundi	558 (421-750)	10.8 (8.25-14.36)	359 (271-479)	7.81 (6.05-10.28)	12,539 (9,388-17,005)	222.3 (167.05-298.01)
Cambodia	2,084 (1,626-2,744)	15.91 (12.53-20.59)	1,085 (869-1,383)	8.9 (7.26-11.15)	36,028 (28,042-46,894)	267.48 (210.97-345.82)
Cameroon	1,908 (1,232-2,673)	13.58 (8.91-19.11)	1,145 (760-1,612)	9.05 (6-12.75)	38,607 (25,050-53,972)	259.35 (171.04-364.98)
Canada	25,021 (22,376-27,959)	42.64 (38.13-47.75)	6,022 (5,515-6,570)	9.17 (8.39-10.02)	143,230 (129,492-158,429)	246.63 (222.3-273.52)
Cape Verde	65 (55-76)	13.94 (11.9-16.17)	30 (26-34)	6.7 (5.75-7.57)	871 (745-1,035)	186.94 (160.01-220.27)
Central African Republic	394 (224-660)	15.31 (9.49-24.23)	284 (166-468)	11.99 (7.78-18.51)	9,784 (5,414-16,655)	346.26 (204.41-571.59)
Chad	616 (428-934)	9.5 (6.68-14.59)	406 (285-617)	6.76 (4.82-10.38)	13,557 (9,339-20,407)	197.3 (137.46-300.87)
Chile	5,053 (4,226-5,969)	22.29 (18.57-26.27)	1,660 (1,417-1,929)	7.18 (6.13-8.33)	42,163 (35,637-49,622)	185.78 (156.95-218.5)
China	363,857 (304,358-394,316)	18.22 (15.18-19.74)	87,564 (73,262-94,156)	4.49 (3.76-4.83)	2638,325 (2222,121-2860,792)	130.62 (110.02-141.64)
Colombia	11,143 (9,399-	20.68 (17.43-24.38)	3,349 (2,888-3,926)	6.18 (5.34-7.25)	96,812 (82,311-114,509)	179.6 (153.19-212.33)

	13,166)					
Comoros	91 (63-122)	17.34 (12.1-23.09)	55 (39-72)	11.21 (7.9-14.69)	1,824 (1,267-2,430)	332.22 (232.3-440.52)
Congo	656 (400-1,019)	20.83 (13.85-30.15)	382 (248-569)	13.61 (9.65-18.87)	13,185 (7,873-20,735)	392.41 (253.09-583.04)
Costa Rica	1,533 (1,359-1,713)	30.49 (27.04-34.09)	423 (384-469)	8.49 (7.7-9.4)	11,615 (10,467-12,928)	229.44 (206.73-255.24)
Cote d'Ivoire	1,876 (1,323-2,488)	14.26 (10.19-18.66)	1,185 (845-1,558)	10.01 (7.17-13.06)	41,176 (28,806-54,592)	290.72 (205.91-382.32)
Croatia	2,717 (2,440-3,030)	35.67 (31.88-39.82)	965 (881-1,061)	11.15 (10.2-12.26)	20,018 (18,173-22,138)	260.75 (235.8-289.54)
Cuba	5,417 (4,602-6,361)	30.28 (25.68-35.78)	1,730 (1,491-2,008)	9.31 (8.01-10.82)	42,887 (36,678-50,253)	243.66 (207.88-283.95)
Cyprus	821 (677-979)	46.75 (38.55-55.86)	172 (147-197)	9.36 (8.02-10.78)	4,717 (3,948-5,534)	268.55 (225.2-316.42)
Czech Republic	5,919 (5,295-6,591)	32.36 (29.01-36)	1,725 (1,572-1,881)	8.52 (7.79-9.29)	38,040 (34,530-41,658)	207.51 (188.04-227.65)
Democratic Republic of the Congo	4,985 (3,560-6,993)	12.42 (9.21-16.74)	3,199 (2,347-4,397)	8.8 (6.64-11.74)	104,208 (73,594-147,361)	241.1 (176.09-331.76)
Denmark	4,619 (4,119-5,128)	47.34 (42.29-52.82)	1,308 (1,188-1,441)	11.73 (10.65-12.93)	27,896 (25,125-31,017)	284.59 (256.29-316.47)
Djibouti	116 (75-172)	15.08 (10.02-21.71)	65 (42-94)	9.66 (6.53-13.74)	2,339 (1,500-3,439)	283.13 (185.05-411.56)
Dominica	27 (23-30)	31.08 (26.93-35.18)	11 (10-13)	12.53 (11.09-14)	285 (251-321)	338.15 (298.03-379.75)
Dominican Republic	2,171 (1,647-2,713)	22.06 (16.8-27.48)	798 (621-986)	8.34 (6.52-10.3)	25,398 (19,040-31,610)	254.82 (192.58-316.56)
Ecuador	2,338 (2,019-2,737)	15.28 (13.21-17.87)	877 (765-1,009)	5.87 (5.12-6.74)	25,799 (22,269-30,055)	166.89 (144.19-194.09)
Egypt	10,543 (8,147-15,846)	13.79 (10.55-22.19)	3,585 (2,755-5,733)	5.14 (3.92-8.85)	136,054 (105,125-202,949)	170.48 (131.15-264.41)
El Salvador	1,122 (863-1,446)	19.69 (15.15-25.37)	376 (296-475)	6.61 (5.21-8.33)	11,012 (8,478-14,164)	193.77 (149.12-249.09)
Equatorial Guinea	113 (66-178)	19.18 (11.41-30.19)	52 (31-83)	10.25 (6.31-15.92)	1,773 (1,026-2,864)	289.57 (171.95-463.34)
Eritrea	578 (413-782)	20.35 (14.95-26.58)	366 (266-484)	14.71 (10.9-18.96)	12,855 (9,218-17,301)	409.82 (299.43-539.09)
Estonia	688 (571-828)	30.81 (25.42-37.37)	221 (186-263)	8.7 (7.26-10.32)	5,038 (4,200-6,055)	229.9 (192.03-276.95)
Ethiopia	6,659 (5,562-8,364)	13.02 (11.02-16.43)	3,898 (3,283-4,906)	8.62 (7.3-10.7)	137,737 (115,117-174,392)	254.51 (214.02-323.46)
Federated States of Micronesia	18 (11-27)	23.04 (15.72-32.72)	9 (6-13)	13.44 (10.05-17.88)	306 (188-453)	361.92 (236.22-521.65)

Fiji	255 (117-328)	31.49 (14.38-40.43)	120 (56-154)	16.7 (7.7-21.21)	3,796 (1,745-4,922)	448.27 (206.42-576.41)
Finland	4,348 (3,876-4,899)	43.87 (39.08-49.59)	925 (838-1,022)	8.08 (7.31-8.92)	21,392 (19,228-23,867)	219.36 (197.09-245.21)
France	48,541 (43,736-54,263)	43.52 (39.01-48.81)	13,603 (12,437-14,830)	10.08 (9.18-11.03)	287,860 (258,582-318,922)	266.56 (238.86-295.93)
Gabon	208 (147-272)	17.48 (12.48-22.5)	117 (84-150)	10.76 (7.8-13.62)	3,681 (2,613-4,845)	295.36 (210.49-385.75)
Georgia	1,863 (1,684-2,038)	35.25 (31.99-38.48)	765 (706-824)	13.54 (12.51-14.55)	20,831 (19,119-22,633)	393.8 (361.62-427.84)
Germany	76,435 (64,921-88,816)	49.04 (41.34-57.41)	20,302 (17,450-23,393)	11.04 (9.46-12.84)	437,750 (372,527-512,254)	285.54 (242.45-336.13)
Ghana	3,189 (2,426-4,000)	16.73 (12.75-20.9)	1,695 (1,291-2,113)	9.93 (7.61-12.29)	57,789 (43,586-72,748)	289.78 (219.95-363.11)
Greece	8,025 (7,166-8,936)	42.13 (37.62-46.96)	2,342 (2,122-2,560)	10.11 (9.18-11.02)	49,863 (44,855-54,949)	266.95 (240.07-294.68)
Greenland	13 (11-19)	18.76 (15.3-25.87)	5 (4-7)	7.84 (6.46-10.46)	147 (120-202)	197.94 (162.08-271.23)
Grenada	44 (39-49)	31.62 (28.39-35.26)	20 (18-22)	13.62 (12.34-14.83)	497 (449-547)	374.35 (337.84-413.04)
Guam	50 (42-58)	27.74 (23.19-32.27)	17 (15-19)	9.64 (8.24-11.1)	495 (420-572)	269.25 (228.41-310.42)
Guatemala	1,259 (1,062-1,466)	10.85 (9.18-12.64)	525 (450-603)	4.75 (4.1-5.45)	16,286 (13,806-19,071)	136.24 (115.82-159.26)
Guinea	803 (558-1,016)	12.94 (9.06-16.41)	524 (369-664)	9 (6.39-11.39)	17,159 (11,886-21,830)	266.38 (185.69-341.88)
Guinea-Bissau	131 (90-183)	14.86 (10.45-20.61)	84 (58-116)	10.65 (7.58-14.53)	2,924 (1,994-4,058)	310.35 (214.69-430.88)
Guyana	134 (111-161)	19.87 (16.56-23.79)	63 (53-74)	9.77 (8.25-11.53)	1,976 (1,632-2,388)	281.75 (234.26-338.29)
Haiti	1,495 (868-2,416)	19.06 (11.52-29.96)	900 (535-1,419)	12.43 (7.85-18.89)	29,623 (16,757-48,500)	351.66 (206.06-559.19)
Honduras	886 (607-1,524)	13.8 (9.54-22.77)	401 (278-647)	6.63 (4.58-10.22)	11,568 (7,964-21,167)	175.19 (122.14-308.09)
Hungary	5,829 (5,240-6,462)	34.31 (30.85-38.07)	2,072 (1,907-2,244)	10.99 (10.13-11.94)	46,573 (42,476-51,064)	275.12 (251.68-301.56)
Iceland	186 (166-206)	38.54 (34.43-42.58)	38 (35-42)	7.3 (6.7-7.94)	946 (852-1,041)	198.79 (179.63-218.28)
India	155,431 (137,711-168,431)	13.02 (11.54-14.08)	80,224 (70,868-86,656)	7.15 (6.31-7.76)	2523,239 (2231,085-2728,163)	205.46 (181.72-221.91)
Indonesia	40,114 (36,180-45,554)	15.53 (14.02-18.22)	17,519 (15,828-21,026)	7.42 (6.69-9.37)	624,430 (562,468-705,505)	234.72 (211.96-270.68)
Iran	16,160 (12,522-	20.01 (15.66-21.84)	4,310 (3,428-4,545)	5.71 (4.55-6.03)	143,767 (113,290-	172.75 (136.14-183.88)

	17,739)				153,096)	
Iraq	3,642 (3,121-4,340)	12.4 (10.7-14.77)	1,490 (1,296-1,749)	5.5 (4.81-6.47)	53,620 (46,218-63,526)	176.09 (153.14-206.99)
Ireland	3,293 (2,899-3,750)	48.7 (42.79-55.39)	755 (674-841)	10.57 (9.44-11.78)	18,778 (16,689-21,275)	278.48 (247.53-315.7)
Israel	4,052 (3,603-4,533)	39.09 (34.77-43.81)	1,209 (1,092-1,325)	10.82 (9.75-11.88)	28,531 (25,688-31,583)	279.9 (251.65-310.69)
Italy	50,106 (44,734-55,559)	44.17 (39.54-49.27)	13,013 (11,800-14,174)	9.2 (8.32-10.05)	276,178 (248,289-304,534)	244.78 (219.21-270.68)
Jamaica	1,020 (804-1,278)	34.83 (27.45-43.73)	399 (322-492)	13.54 (10.93-16.7)	11,349 (9,097-14,198)	388.44 (311.3-485.84)
Japan	72,067 (66,799-77,249)	29.21 (27.11-31.33)	14,512 (13,915-15,118)	4.96 (4.75-5.17)	353,757 (331,486-377,047)	155.4 (145.63-165.59)
Jordan	1,744 (1,340-2,229)	23.77 (18.3-30.03)	491 (382-615)	7.52 (5.83-9.41)	16,729 (12,943-21,204)	216.56 (167.4-271.3)
Kazakhstan	3,791 (3,359-4,218)	20.69 (18.39-22.91)	1,380 (1,244-1,516)	8 (7.23-8.73)	41,223 (36,992-45,523)	221.34 (198.92-243.65)
Kenya	3,409 (2,720-4,161)	12.72 (10.24-15.43)	1,735 (1,385-2,111)	7.4 (5.95-8.93)	60,037 (47,616-73,445)	213.02 (170.28-259.31)
Kiribati	14 (10-19)	17.59 (12.7-22.9)	9 (6-11)	12.21 (9.03-15.69)	288 (206-383)	337.32 (243.49-438.59)
Kuwait	582 (495-667)	14.88 (12.74-16.95)	118 (103-131)	3.58 (3.12-3.98)	4,464 (3,867-5,042)	108.54 (94.06-121.91)
Kyrgyzstan	630 (565-700)	12.37 (11.19-13.64)	259 (236-282)	5.53 (5.07-5.99)	8,181 (7,458-8,963)	157.51 (144.1-172.42)
Laos	733 (523-1,002)	13.75 (10.07-18.49)	373 (274-502)	7.71 (5.78-10.2)	13,309 (9,578-18,274)	242.46 (176.66-330.6)
Latvia	1,056 (884-1,254)	30.28 (25.19-36.04)	396 (334-465)	10.32 (8.67-12.22)	9,166 (7,704-10,971)	275.03 (229.26-329.98)
Lebanon	4,976 (3,826-6,139)	74.09 (57.3-91.38)	977 (769-1,189)	15.7 (12.32-18.96)	32,118 (24,907-39,650)	472.62 (365.4-584.29)
Lesotho	222 (148-314)	17.91 (12.15-24.74)	148 (100-205)	13.03 (9.12-17.6)	4,288 (2,801-6,182)	323.5 (213.26-459.13)
Liberia	294 (210-410)	11.87 (8.61-16.27)	177 (128-245)	7.98 (5.79-10.83)	6,036 (4,290-8,413)	228.65 (164.44-316.42)
Libya	1,525 (1,173-1,922)	25.18 (19.72-31.49)	411 (322-508)	7.57 (6-9.31)	14,947 (11,558-18,821)	237.15 (183.52-294.41)
Lithuania	1,467 (1,311-1,637)	30.61 (27.16-34.16)	529 (479-584)	9.68 (8.74-10.7)	12,727 (11,444-14,145)	267.53 (239.74-297.85)
Luxembourg	450 (375-529)	50.15 (41.71-58.83)	106 (90-122)	10.85 (9.24-12.53)	2,454 (2,046-2,845)	275.1 (229.16-319.98)
Macedonia	921 (774-1,079)	29.53 (24.66-34.59)	304 (257-352)	9.29 (7.88-10.72)	8,784 (7,350-10,223)	279.44 (234.35-324.06)
Madagascar	1,693 (1,283-2,204)	12.23 (9.22-15.75)	1,022 (776-1,322)	8.26 (6.27-10.54)	36,987 (28,244-48,023)	249.49 (190.24-321.62)
Malawi	781 (507-1,074)	9.11 (5.93-12.4)	491 (325-665)	6.2 (4.1-8.34)	15,643 (10,281-21,555)	174.95 (115.17-240.74)

Malaysia	8,183 (6,006-9,855)	28.58 (20.71-34.18)	2,592 (1,845-3,062)	9.82 (6.82-11.59)	82,656 (60,059-98,683)	284.88 (204.41-338.96)
Maldives	44 (36-54)	12 (9.96-14.27)	13 (11-15)	4.09 (3.39-4.87)	415 (345-497)	113.36 (94.35-133.58)
Mali	1,163 (796-1,590)	11.73 (8.09-15.72)	675 (463-907)	7.36 (5.11-9.75)	22,739 (15,478-31,178)	220.72 (151.47-298.93)
Malta	382 (340-426)	50.12 (44.75-55.57)	101 (92-112)	11.99 (10.93-13.16)	2,350 (2,125-2,601)	312.94 (281.95-346.3)
Marshall Islands	11 (7-17)	25.5 (17.34-37.4)	5 (3-7)	13.49 (9.78-19.05)	182 (117-269)	403.01 (269.91-588.88)
Mauritania	350 (268-449)	15.29 (11.73-19.73)	190 (145-244)	8.92 (6.85-11.51)	6,049 (4,638-7,730)	257.39 (197.54-329.95)
Mauritius	556 (490-628)	33.21 (29.22-37.36)	182 (163-202)	10.94 (9.78-12.09)	5,486 (4,887-6,143)	320.39 (284.57-357.29)
Mexico	25,600 (24,755-26,451)	20.95 (20.27-21.64)	7,624 (7,395-7,846)	6.45 (6.25-6.63)	236,703 (228,448-246,199)	190.11 (183.48-197.73)
Moldova	1,246 (1,122-1,360)	22.67 (20.44-24.77)	510 (470-547)	9.02 (8.34-9.66)	14,099 (12,974-15,216)	255.78 (235.81-275.93)
Mongolia	262 (209-341)	9.49 (7.72-11.68)	117 (95-145)	4.88 (4.03-5.85)	3,947 (3,138-5,162)	136.13 (110.5-169.24)
Montenegro	333 (271-406)	36.64 (29.73-44.64)	106 (89-126)	11 (9.23-13.13)	2,780 (2,278-3,387)	302.25 (247.84-368.48)
Morocco	8,809 (6,323-11,735)	24.59 (17.9-32.5)	3,218 (2,387-4,228)	9.22 (6.89-12.05)	115,087 (80,798-155,117)	315.34 (224.56-424.51)
Mozambique	2,090 (1,491-2,769)	15.11 (10.89-19.69)	1,281 (923-1,674)	10.61 (7.77-13.65)	45,587 (32,325-60,495)	299.24 (214.88-390.83)
Myanmar	12,866 (10,053-16,162)	25 (19.62-31.24)	6,327 (4,961-7,893)	12.98 (10.26-16.2)	214,590 (167,981-270,483)	408.29 (320.68-510.82)
Namibia	369 (258-502)	23.66 (16.8-32.08)	202 (147-271)	13.83 (10.22-18.37)	6,108 (4,154-8,312)	381.2 (262.57-516.54)
Nepal	2,718 (1,943-4,996)	11.54 (8.33-21.22)	1,535 (1,123-2,803)	6.88 (5.07-12.66)	48,475 (35,211-88,091)	200.55 (145.63-362.3)
Netherlands	16,415 (14,512-18,333)	57.29 (50.84-63.96)	3,914 (3,556-4,267)	12.04 (10.97-13.08)	89,194 (79,983-98,099)	313.58 (280.9-345.01)
New Zealand	3,039 (2,735-3,398)	46.15 (41.49-51.42)	737 (672-805)	10.32 (9.42-11.25)	19,428 (17,633-21,407)	302.21 (273.88-332.22)
Nicaragua	716 (583-861)	14.4 (11.69-17.24)	245 (200-290)	5.15 (4.22-6.09)	7,448 (6,041-8,953)	146.41 (119.19-175.88)
Niger	742 (527-1,127)	8.39 (6.03-12.73)	475 (341-720)	5.85 (4.28-8.92)	15,771 (11,220-23,897)	168.2 (120.51-256.3)
Nigeria	30,070 (19,104-44,529)	28.61 (18.42-42.04)	15,759 (10,159-23,109)	16.65 (11.01-23.9)	525,464 (331,339-786,071)	481.73 (308.04-710.78)

North Korea	5,081 (3,571-6,854)	15.58 (11-20.9)	2,160 (1,554-2,819)	6.75 (4.87-8.83)	67,779 (47,321-91,595)	204.65 (144.27-275.65)
Northern Mariana Islands	16 (13-20)	28.54 (23.74-34.52)	5 (4-6)	9.37 (8.02-10.9)	146 (119-177)	246.06 (204.82-295.41)
Norway	3,123 (2,914-3,354)	38.02 (35.44-40.9)	741 (707-772)	8.01 (7.62-8.36)	17,087 (15,980-18,147)	210.09 (196.48-223.57)
Oman	391 (298-492)	13.3 (10.27-16.34)	90 (70-111)	3.88 (3.06-4.6)	3,321 (2,552-4,197)	104.64 (80.46-128.07)
Pakistan	38,603 (24,290-77,685)	27.66 (17.32-56.49)	20,241 (12,546-41,300)	16.59 (10.19-34.51)	691,900 (437,327-1388,105)	477.68 (297.55-970.41)
Palestine	702 (587-811)	24.38 (20.43-28.16)	269 (226-309)	10.22 (8.59-11.81)	8,762 (7,392-10,065)	287.83 (242.3-331.27)
Panama	905 (804-1,016)	22.51 (20.06-25.26)	274 (247-304)	6.83 (6.14-7.58)	7,835 (6,993-8,745)	194.83 (174.03-217.67)
Papua New Guinea	1,086 (704-1,694)	17.43 (12.45-25.6)	545 (375-813)	10.34 (7.9-14.45)	21,143 (13,828-32,966)	322.85 (225.41-480.22)
Paraguay	1,186 (886-1,514)	20.89 (15.7-26.6)	491 (371-620)	9.04 (6.85-11.31)	14,479 (10,846-18,621)	249.93 (187.79-320.87)
Peru	4,489 (3,563-5,739)	14.24 (11.32-18.12)	1,683 (1,343-2,093)	5.41 (4.32-6.7)	48,961 (38,808-62,114)	155.27 (123.22-196.96)
Philippines	23,330 (18,688-28,503)	27.57 (22.3-33.81)	9,930 (8,147-12,191)	12.94 (10.72-15.7)	330,500 (267,133-408,160)	380.43 (309.64-466.86)
Poland	19,323 (17,284-21,573)	31.21 (28.05-34.92)	6,260 (5,715-6,861)	9.27 (8.47-10.18)	150,631 (136,580-165,668)	243.65 (221.05-267.97)
Portugal	6,897 (6,122-7,777)	36.7 (32.44-41.58)	1,965 (1,782-2,168)	8.64 (7.81-9.53)	43,762 (39,301-48,600)	234.4 (209.85-260.7)
Puerto Rico	2,023 (1,786-2,280)	34.71 (30.72-39.12)	542 (486-598)	8.41 (7.53-9.29)	13,613 (12,172-15,106)	244.22 (218.12-272.18)
Qatar	334 (256-423)	23.13 (17.96-28.91)	67 (52-84)	6.16 (4.87-7.59)	2,560 (1,988-3,239)	153.78 (120.16-192.76)
Romania	9,138 (8,279-10,116)	28.51 (25.79-31.63)	3,408 (3,139-3,693)	9.62 (8.85-10.42)	83,641 (76,538-90,987)	261.03 (239.07-283.53)
Russian Federation	70,416 (67,404-73,389)	32.41 (31-33.81)	22,068 (21,546-22,608)	9.67 (9.44-9.91)	593,010 (572,815-613,566)	271.83 (262.35-281.35)
Rwanda	952 (564-1,403)	13.68 (8.34-19.67)	548 (332-792)	8.81 (5.48-12.41)	18,530 (10,924-27,352)	251.1 (150.4-366.12)
Saint Lucia	61 (54-69)	28.66 (25.27-32.21)	24 (21-27)	11.21 (10.09-12.48)	672 (597-759)	311.16 (277.08-350.9)
Saint Vincent and the	39 (35-44)	29.45 (26.21-33.03)	17 (15-19)	12.46 (11.24-13.75)	490 (437-546)	365.81 (326.02-406.71)

Grenadines						
Samoa	20 (14-26)	13.23 (9.42-17.14)	10 (7-12)	6.79 (5.03-8.83)	302 (208-392)	195.48 (136.35-252.35)
Sao Tome and Principe	20 (13-26)	15.74 (10-21.08)	11 (7-14)	9.26 (5.92-12.45)	362 (229-478)	272.77 (173.52-364.14)
Saudi Arabia	5,234 (3,920-6,975)	18.33 (14.22-23.64)	905 (702-1,201)	3.77 (3.03-4.8)	37,412 (28,050-50,246)	120.37 (93.99-155.53)
Senegal	1,109 (819-1,574)	13.4 (9.93-19.05)	703 (520-998)	9.09 (6.78-12.83)	22,546 (16,542-32,077)	260.45 (191.1-372.05)
Serbia	5,616 (4,952-6,432)	40.4 (35.61-46.19)	2,222 (1,962-2,479)	14.74 (13.08-16.47)	51,473 (45,677-58,340)	366.31 (323.77-415.43)
Seychelles	35 (28-40)	29.87 (24.12-33.92)	12 (10-14)	10.96 (8.7-12.27)	370 (301-419)	308.87 (250.61-349.4)
Sierra Leone	552 (397-757)	13.4 (9.72-18.46)	339 (245-466)	8.99 (6.59-12.31)	11,309 (8,106-15,583)	260.88 (187.16-360.02)
Singapore	2,046 (1,809-2,306)	28.45 (25.15-32.08)	396 (359-440)	5.59 (5.06-6.2)	12,438 (11,129-13,929)	170.88 (152.83-191.75)
Slovakia	2,878 (2,405-3,304)	33.9 (28.65-39.07)	909 (720-1,025)	10.29 (8.28-11.57)	22,012 (18,549-25,076)	258.07 (221.61-293.8)
Slovenia	1,181 (1,046-1,344)	32.82 (28.9-37.52)	388 (346-434)	9.13 (8.15-10.24)	8,109 (7,194-9,133)	220.24 (195.04-248.86)
Solomon Islands	64 (43-101)	15.44 (11.31-23.35)	35 (25-52)	9.57 (7.46-13.67)	1,240 (813-1,968)	284.54 (200.41-437.54)
Somalia	939 (672-1,291)	11.96 (8.7-16.4)	640 (456-875)	9.07 (6.72-12.26)	22,324 (15,688-30,939)	264.47 (189.64-363.78)
South Africa	8,287 (7,292-9,398)	17.51 (15.42-19.39)	4,151 (3,647-4,575)	9.51 (8.33-10.44)	119,074 (106,334-139,196)	243.53 (215.33-278.36)
South Korea	17,622 (15,397-20,033)	21.64 (19-24.49)	3,633 (3,256-3,999)	4.34 (3.89-4.78)	106,235 (94,367-118,128)	128.41 (114.02-142.43)
South Sudan	527 (372-766)	10.45 (7.45-15.08)	324 (228-466)	7.19 (5.16-10.29)	11,726 (8,143-17,008)	216.6 (152.64-311.6)
Spain	26,772 (24,006-29,816)	33.09 (29.71-36.89)	6,908 (6,356-7,556)	7.21 (6.65-7.88)	155,099 (141,323-170,387)	196.5 (178.35-216.94)
Sri Lanka	4,070 (2,863-5,354)	16.25 (11.48-21.23)	1,314 (917-1,696)	5.42 (3.78-6.94)	38,744 (26,981-51,052)	151.93 (106.76-198.75)
Sudan	2,191 (1,320-3,639)	9.44 (5.97-15.28)	982 (612-1,596)	4.61 (3.05-7.25)	35,113 (20,712-58,568)	145.17 (88.68-239.14)
Suriname	111 (95-129)	18.49 (15.92-21.46)	51 (44-58)	8.69 (7.59-9.89)	1,459 (1,253-1,686)	236.96 (204.04-274.04)
Swaziland	109 (71-158)	17.65 (11.89-24.8)	62 (42-88)	11.57 (8.01-15.91)	1,943 (1,267-2,825)	291.26 (193.27-415.95)
Sweden	7,953 (7,264-8,749)	46.42 (42.45-50.93)	1,804 (1,669-1,955)	9.02 (8.34-9.79)	39,884 (36,552-43,836)	238.72 (218.17-261.6)

Switzerland	5,715 (5,076-6,384)	38.39 (34.05-43.02)	1,462 (1,329-1,617)	8.64 (7.85-9.53)	32,178 (28,887-35,778)	219.42 (196.58-244.11)
Syria	2,274 (1,704-2,911)	14.9 (11.27-18.77)	690 (529-864)	4.76 (3.7-5.88)	23,844 (18,009-30,595)	150.73 (114.78-191.7)
Taiwan	11,344 (10,169-12,718)	31.6 (28.24-35.32)	2,324 (2,131-2,542)	6.18 (5.67-6.76)	73,105 (66,562-80,267)	200.69 (182.47-220.69)
Tajikistan	745 (609-877)	10.97 (8.95-12.81)	321 (265-372)	5.28 (4.26-6.11)	11,469 (9,530-13,418)	164.7 (136.68-192.29)
Tanzania	3,648 (2,796-4,823)	12.44 (9.69-16.16)	2,120 (1,660-2,764)	8.08 (6.45-10.25)	71,594 (54,800-94,913)	231.16 (177.88-306.48)
Thailand	18,213 (14,241-21,701)	18.45 (14.42-22.04)	5,656 (4,419-6,588)	5.68 (4.44-6.62)	176,944 (140,744-207,390)	176.58 (139.73-207.25)
The Bahamas	214 (183-248)	52.29 (44.93-60.18)	72 (62-82)	18.36 (16-20.84)	2,283 (1,960-2,606)	539.62 (463.45-613.54)
The Gambia	108 (72-142)	10.19 (6.91-13.32)	66 (45-86)	6.69 (4.54-8.74)	2,078 (1,397-2,747)	187.88 (127-246.41)
Timor-Leste	98 (59-141)	11.36 (7-16.12)	49 (32-69)	5.99 (3.95-8.31)	1,576 (933-2,244)	181.01 (110.76-256.26)
Togo	589 (434-779)	13.68 (10.18-17.94)	352 (261-462)	9.18 (6.86-11.9)	11,923 (8,806-15,715)	259.04 (191.36-340.78)
Tonga	29 (23-37)	35.13 (27.57-43.97)	14 (11-18)	18.23 (14.41-22.54)	411 (319-519)	480.88 (376.7-606.01)
Trinidad and Tobago	521 (385-690)	29.13 (21.57-38.85)	200 (150-257)	11.25 (8.49-14.51)	5,607 (4,139-7,377)	312.68 (230.39-413.46)
Tunisia	2,906 (2,094-3,964)	22.66 (16.33-30.78)	820 (599-1,106)	6.58 (4.85-8.86)	25,455 (18,547-34,521)	195.08 (142.9-263.79)
Turkey	16,976 (14,425-20,819)	18.76 (15.96-23)	4,549 (3,924-5,522)	5.07 (4.37-6.16)	145,404 (124,685-176,619)	159.22 (136.83-193.39)
Turkmenistan	883 (760-1,014)	18.97 (16.51-21.66)	322 (283-365)	7.62 (6.72-8.6)	11,224 (9,810-12,802)	236.19 (206.52-268.61)
Uganda	2,382 (1,738-3,167)	14.21 (10.5-18.68)	1,441 (1,062-1,899)	9.59 (7.21-12.43)	48,814 (35,438-65,068)	275.24 (202.25-363.8)
Ukraine	18,824 (17,109-20,711)	27.17 (24.65-29.98)	7,716 (7,130-8,377)	10.45 (9.63-11.36)	211,374 (193,547-231,397)	306.21 (278.67-335.09)
United Arab Emirates	1,123 (821-1,483)	17.39 (13.12-22.16)	291 (216-375)	6.06 (4.59-7.65)	11,634 (8,589-15,154)	156.04 (118.13-196.45)
United Kingdom	58,163 (56,519-59,902)	53.88 (52.32-55.55)	13,988 (13,708-14,297)	11.33 (11.1-11.59)	311,819 (297,993-327,549)	298.45 (285.02-313.4)
United States	251,858 (242,536-262,409)	50.39 (48.42-52.56)	49,390 (47,718-51,117)	9.27 (8.95-9.59)	1262,965 (1193,321-1339,994)	260.96 (245.92-276.97)

Uruguay	1,875 (1,565-2,192)	39.26 (32.55-46.36)	778 (657-903)	14.66 (12.34-16.95)	17,583 (14,672-20,575)	380.04 (316.53-447.01)
Uzbekistan	5,045 (4,194-6,038)	17.8 (14.87-21.14)	1,779 (1,509-2,121)	7.04 (6.01-8.32)	62,844 (53,108-75,265)	217.04 (184.58-258.77)
Vanuatu	41 (27-62)	21 (13.94-30.98)	23 (15-33)	13.06 (8.88-18.56)	785 (507-1,184)	386.82 (254.19-573.61)
Venezuela	8,259 (6,596-10,161)	27.56 (22.14-33.88)	2,465 (2,004-3,022)	8.57 (7-10.49)	74,631 (59,874-91,838)	243.53 (195.19-299.43)
Vietnam	15,099 (11,566-19,021)	14.82 (11.43-18.47)	5,762 (4,524-7,068)	6.03 (4.72-7.33)	178,718 (138,664-227,114)	172.19 (133.74-216.02)
Virgin Islands, U.S.	74 (63-87)	44.83 (37.82-53.41)	25 (21-28)	14.45 (12.38-16.92)	622 (531-725)	379.99 (321.06-449.82)
Yemen	1,744 (1,078-2,875)	10.59 (6.9-16.92)	824 (529-1,324)	5.51 (3.73-8.64)	29,392 (17,841-49,226)	171.17 (107.7-279.83)
Zambia	1,178 (913-1,500)	14.16 (11.12-17.97)	716 (562-907)	9.83 (7.85-12.31)	25,273 (19,748-31,851)	280.97 (219.15-358.15)
Zimbabwe	1,194 (754-1,548)	15.06 (10.52-19.02)	761 (501-968)	10.5 (7.75-13.07)	24,821 (14,682-32,030)	293.71 (187.93-374.61)

Abbreviations: ASIR, age-standardized incident rates. ASMR, age-standardized mortality rates. DALY, disability adjusted life-year. SDI, socio-demographic index. UI, uncertain interval.

	Both			Female		
	Incidence	Mortality	DALYs	Incidence	Mortality	DALYs
**Global**	2	5	5	1	1	1
**High SDI**	3	4	3	1	2	2
**High-middle SDI**	3	6	5	1	2	2
**Middle SDI**	2	6	4	1	2	1
**Low-middle SDI**	1	2	1	1	1	1
**Low SDI**	2	3	2	2	1	1
**Central Asia**	1	4	3	1	1	1
Armenia	1	4	2	1	1	1
Azerbaijan	1	6	3	1	1	1
Georgia	1	3	2	1	1	1
Kazakhstan	1	4	4	1	1	1
Kyrgyzstan	1	5	4	1	1	1
Mongolia	6	8	7	4	5	4
Tajikistan	2	6	5	1	2	1
Turkmenistan	1	4	1	1	1	1
Uzbekistan	1	3	2	1	1	1
**East Asia**	5	6	6	1	4	2
China	5	6	6	1	4	2
North Korea	4	6	5	1	3	1
Taiwan	4	5	4	1	4	2
**South Asia**	1	2	1	1	1	1
Bangladesh	2	4	2	1	1	1
Bhutan	1	3	1	1	1	1
India	1	3	1	1	1	1
Nepal	1	3	2	1	1	1
Pakistan	1	1	1	1	1	1
**Southeast Asia**	1	4	3	1	1	1
Cambodia	2	3	2	1	1	1
Indonesia	1	2	2	1	1	1
Laos	2	4	3	1	1	1
Malaysia	1	3	3	1	1	1
Maldives	1	4	2	1	1	1
Mauritius	1	2	1	1	1	1
Myanmar	1	2	1	1	1	1
Philippines	1	2	1	1	1	1
Seychelles	3	4	4	1	1	1
Sri Lanka	1	2	2	1	1	1
Thailand	3	4	4	1	3	1
Timor-Leste	2	7	4	1	1	1
Vietnam	3	5	5	1	3	2
**High-income Asia Pacific**	4	7	6	1	5	1
Brunei	1	3	3	1	2	1
Japan	4	7	6	1	5	2
Singapore	2	4	3	1	1	1
South Korea	5	7	6	1	4	1
**Australasia**	4	4	3	1	2	1
Australia	4	4	3	1	2	1
New Zealand	3	3	3	1	3	1
**Oceania**	2	3	3	2	1	2
American Samoa	1	2	2	1	1	1
Federated States of Micronesia	1	2	2	1	1	1
Fiji	1	1	1	1	1	1
Guam	2	4	4	1	2	2
Kiribati	2	3	3	2	2	2
Marshall Islands	1	2	2	1	1	1
Northern Mariana Islands	1	2	2	1	1	1
Papua New Guinea	3	4	4	2	2	2
Samoa	1	4	2	1	1	1
Solomon Islands	2	3	3	2	1	2
Tonga	1	3	3	1	1	1
Vanuatu	1	3	3	1	1	1
**High-income North America**	2	3	3	1	2	2
Canada	3	3	3	1	2	2
Greenland	3	6	6	2	3	3
United States	2	3	3	1	2	2
**Caribbean**	2	4	2	1	1	1
Antigua and Barbuda	2	2	2	1	1	1
Barbados	2	3	2	1	1	1
Belize	3	7	8	2	2	2
Bermuda	3	4	4	1	1	1
Cuba	4	4	4	1	2	2
Dominica	2	4	3	1	1	1
Dominican Republic	2	4	3	1	1	1
Grenada	2	3	2	1	1	1
Guyana	2	2	1	1	1	2
Haiti	3	4	2	2	2	2
Jamaica	2	4	2	1	1	1
Puerto Rico	3	4	3	1	1	1
Saint Lucia	2	2	2	1	1	1
Saint Vincent and the Grenadines	2	2	2	1	1	1
Suriname	2	4	3	1	1	2
The Bahamas	1	2	1	1	1	1
Trinidad and Tobago	2	3	2	1	1	1
Virgin Islands, U.S.	3	4	4	1	1	1
**Andean Latin America**	3	7	5	1	3	2
Bolivia	4	7	5	2	3	3
Ecuador	3	7	6	1	3	2
Peru	3	6	5	1	3	2
**Central Latin America**	2	5	4	1	1	1
Colombia	2	5	4	1	1	1
Costa Rica	2	5	3	1	1	1
El Salvador	2	7	5	1	2	2
Guatemala	5	8	6	2	4	4
Honduras	2	5	2	1	1	2
Mexico	2	4	2	1	1	1
Nicaragua	2	6	5	2	2	2
Panama	2	5	2	1	1	1
Venezuela	2	5	2	1	1	1
**Southern Latin America**	1	4	3	1	1	1
Argentina	1	3	3	1	1	1
Chile	3	6	5	1	2	1
Uruguay	1	3	3	1	1	1
**Tropical Latin America**	1	5	2	1	1	1
Brazil	1	5	2	1	1	1
Paraguay	1	4	3	1	1	2
**Central Europe**	3	3	3	1	3	1
Albania	2	8	5	1	1	1
Bosnia and Herzegovina	3	4	3	1	1	1
Bulgaria	2	3	3	1	1	1
Croatia	3	3	3	1	1	1
Czech Republic	3	4	4	1	3	1
Hungary	3	3	3	1	3	2
Macedonia	2	4	4	1	1	1
Montenegro	2	3	3	1	2	1
Poland	3	3	3	1	3	2
Romania	3	4	3	1	1	1
Serbia	3	3	3	1	1	1
Slovakia	3	3	3	1	2	1
Slovenia	4	5	3	1	1	1
**Eastern Europe**	2	4	4	1	1	1
Belarus	4	4	4	1	2	1
Estonia	4	6	4	1	2	1
Latvia	3	4	4	1	1	1
Lithuania	4	4	4	1	1	1
Moldova	2	3	3	1	1	1
Russian Federation	1	4	4	1	1	1
Ukraine	3	4	4	1	1	1
**Western Europe**	2	3	3	1	1	1
Andorra	1	4	3	1	1	1
Austria	2	4	3	1	1	1
Belgium	1	3	3	1	1	1
Cyprus	1	3	2	1	1	1
Denmark	3	4	3	1	2	2
Finland	2	5	3	1	1	1
France	1	3	3	1	1	1
Germany	1	3	3	1	1	1
Greece	1	3	3	1	1	1
Iceland	2	5	4	1	2	2
Ireland	1	3	3	1	2	1
Israel	1	3	2	1	1	1
Italy	2	3	3	1	1	1
Luxembourg	1	3	3	1	1	1
Malta	1	3	3	1	1	1
Netherlands	2	3	3	1	2	2
Norway	3	5	4	1	3	2
Portugal	3	5	4	1	2	1
Spain	3	6	3	1	2	1
Sweden	2	4	3	1	2	1
Switzerland	2	4	3	1	1	1
United Kingdom	1	3	3	1	2	2
**Central Sub-Saharan Africa**	2	2	2	2	2	2
Angola	2	3	3	2	2	2
Central African Republic	2	2	2	2	2	2
Congo	2	2	2	2	2	2
Democratic Republic of the Congo	2	2	2	2	2	2
Equatorial Guinea	2	1	1	2	1	1
Gabon	2	2	1	2	1	1
**Eastern Sub-Saharan Africa**	2	3	5	2	2	2
Burundi	3	4	4	2	2	2
Comoros	2	2	3	2	2	2
Djibouti	2	4	3	2	2	2
Eritrea	2	3	3	2	2	2
Ethiopia	2	2	2	2	1	1
Kenya	2	3	2	2	1	1
Madagascar	2	3	3	2	2	2
Malawi	5	5	5	3	5	5
Mozambique	3	4	4	2	2	2
Rwanda	2	3	3	2	2	2
Somalia	3	5	5	2	2	3
South Sudan	3	8	6	2	2	3
Tanzania	3	4	5	2	2	3
Uganda	4	5	5	2	3	3
Zambia	3	3	4	2	2	2
**North Africa and Middle East**	1	4	3	1	1	1
Afghanistan	3	3	4	1	2	3
Algeria	1	2	1	1	1	1
Bahrain	1	2	1	1	1	1
Egypt	2	3	3	1	1	1
Iran	1	5	4	1	1	1
Iraq	1	2	3	1	1	1
Jordan	1	3	3	1	1	1
Kuwait	1	3	1	1	1	1
Lebanon	1	2	1	1	1	1
Libya	1	3	4	1	1	1
Morocco	1	2	1	1	1	1
Oman	1	6	4	1	1	1
Palestine	1	3	2	1	1	1
Qatar	1	2	3	1	1	1
Saudi Arabia	1	5	3	1	1	1
Sudan	2	5	5	1	1	2
Syria	1	4	3	1	1	1
Tunisia	1	3	2	1	1	1
Turkey	3	5	6	1	1	1
United Arab Emirates	2	6	5	1	2	1
Yemen	2	5	5	1	2	1
**Southern Sub-Saharan Africa**	2	5	3	2	2	2
Botswana	2	3	2	2	1	2
Lesotho	2	4	4	2	2	2
Namibia	1	1	2	1	1	1
South Africa	2	5	3	2	2	2
Swaziland	2	5	5	2	2	2
Zimbabwe	2	6	5	2	2	2
**Western Sub-Saharan Africa**	1	2	2	1	1	1
Benin	3	5	4	2	2	2
Burkina Faso	2	3	3	2	2	2
Cameroon	3	6	3	2	2	2
Cape Verde	3	8	6	1	1	1
Chad	5	6	7	2	2	2
Cote d'Ivoire	2	2	1	1	1	1
Ghana	2	2	2	2	1	1
Guinea	3	4	5	2	3	2
Guinea-Bissau	3	4	3	2	2	2
Liberia	3	5	3	2	2	2
Mali	5	4	4	2	2	3
Mauritania	3	4	3	2	2	2
Niger	5	7	6	2	2	2
Nigeria	1	2	2	1	1	1
Sao Tome and Principe	3	6	3	2	2	3
Senegal	3	6	3	2	2	2
Sierra Leone	2	5	3	2	2	2
The Gambia	3	4	5	2	3	3
Togo	2	4	3	2	2	2

Supplementary Figure 1. Breast cancer ranking by total incidence, mortality and DALYs at global, regional, and national levels in 2017.

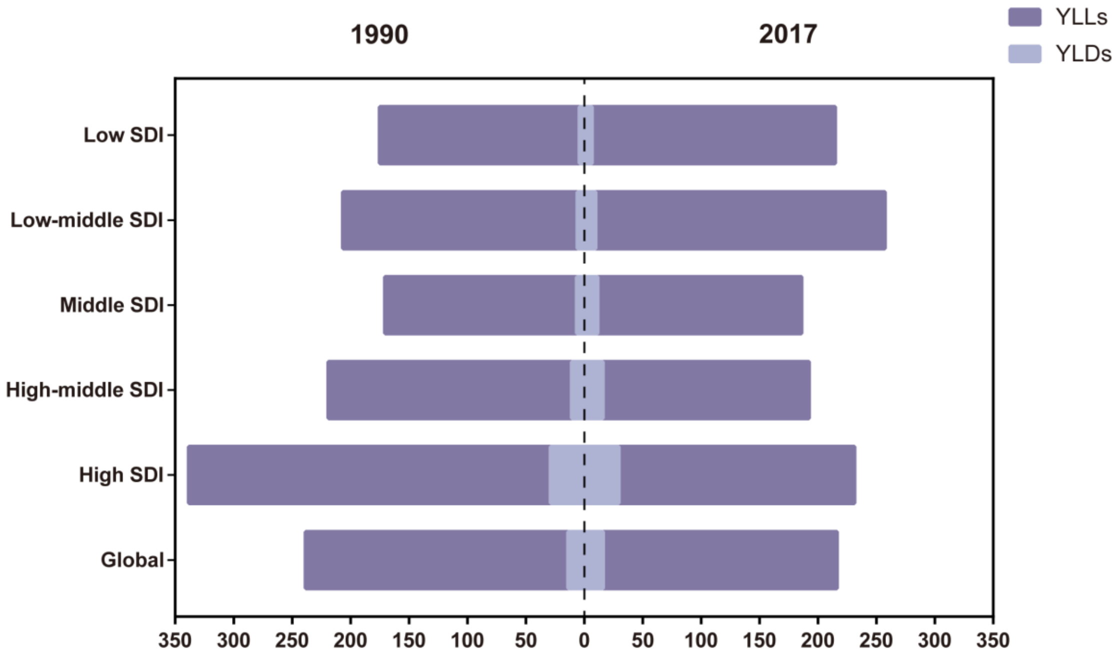

Supplementary Figure 2. Age-standardized DALY rates from breast cancer YLDs and YLLs globally and in 5 SDI quintiles.

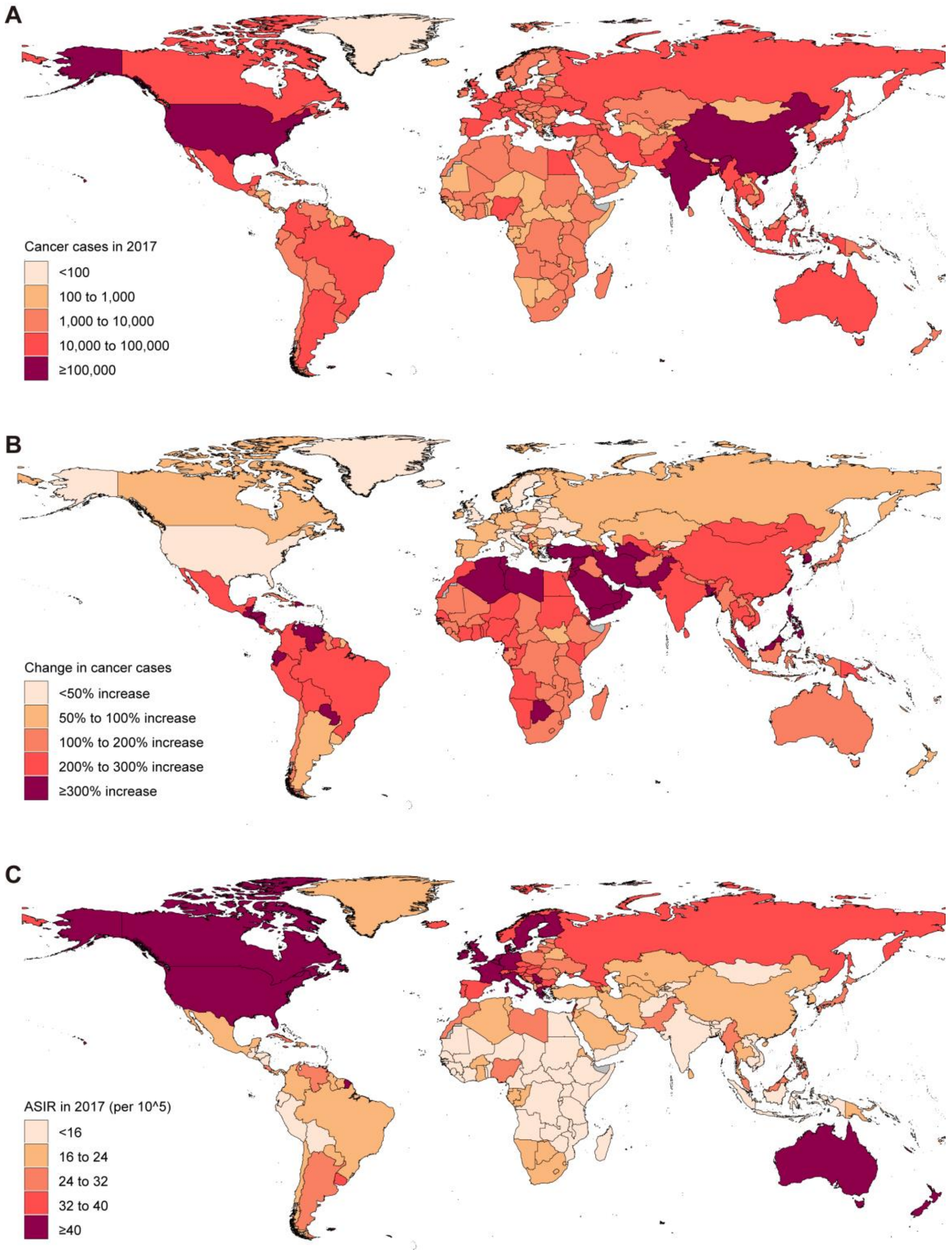

Supplementary Figure 3. The global disease burden of breast cancer incidence in 195 countries and territories. **(A)** The incident cases of breast cancer in 2017. **(B)** The relative change in incident cases of breast cancer between 1990 and 2017. **(C)** The ASIR of breast cancer in 2017.

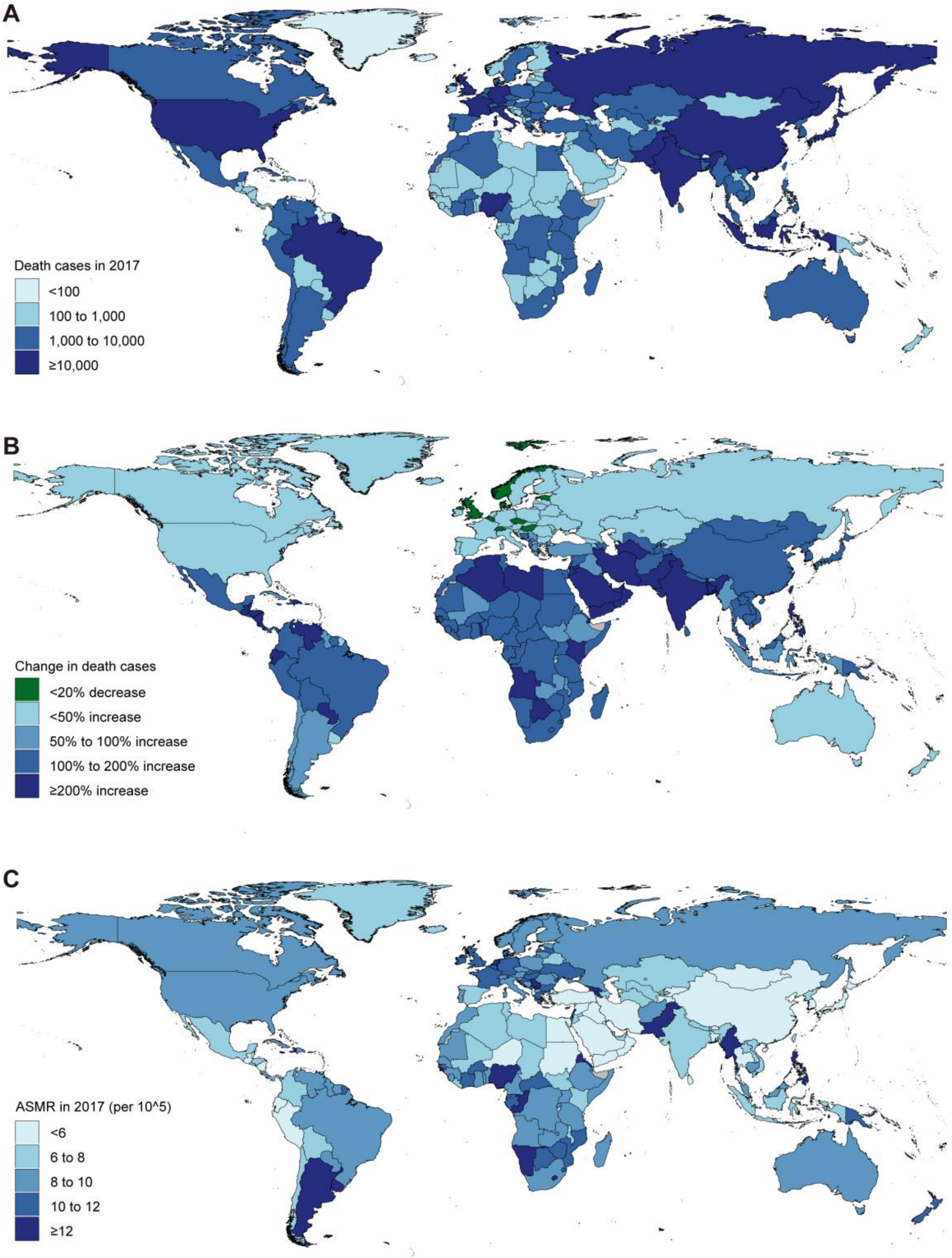

Supplementary Figure 4. The global disease burden of breast cancer mortality in 195 countries and territories. **(A)** The deaths of breast cancer in 2017. **(B)** The relative change in deaths of breast cancer between 1990 and 2017. **(C)** The ASMR of breast cancer in 2017.

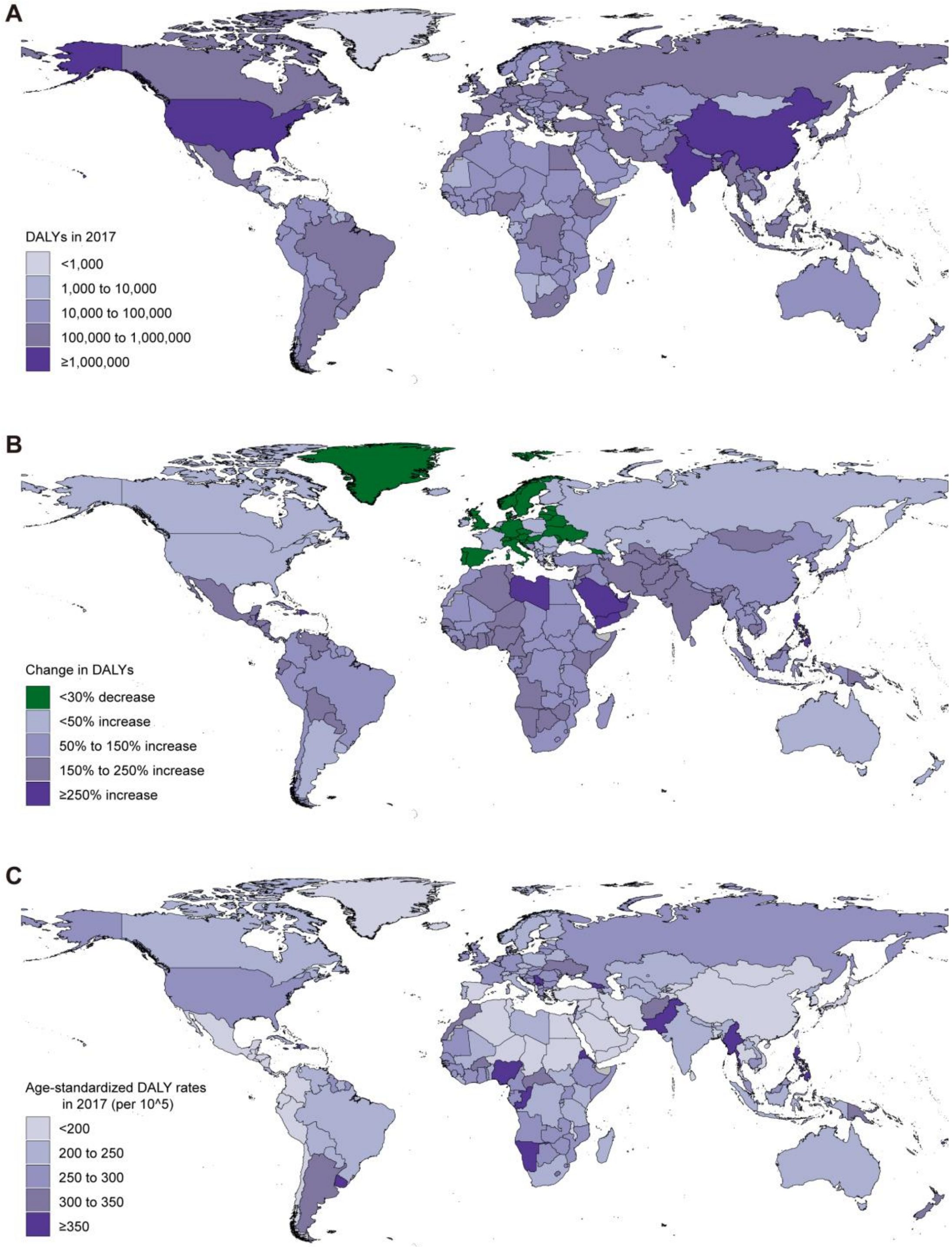

Supplementary Figure 5. The global disease burden of breast cancer DALYs in 195 countries and territories. **(A)** The DALYs of breast cancer in 2017; **(B)** The relative change in DALYs of breast cancer between 1990 and 2017; **(C)** The age-standardized DALY rates of breast cancer in 2017.

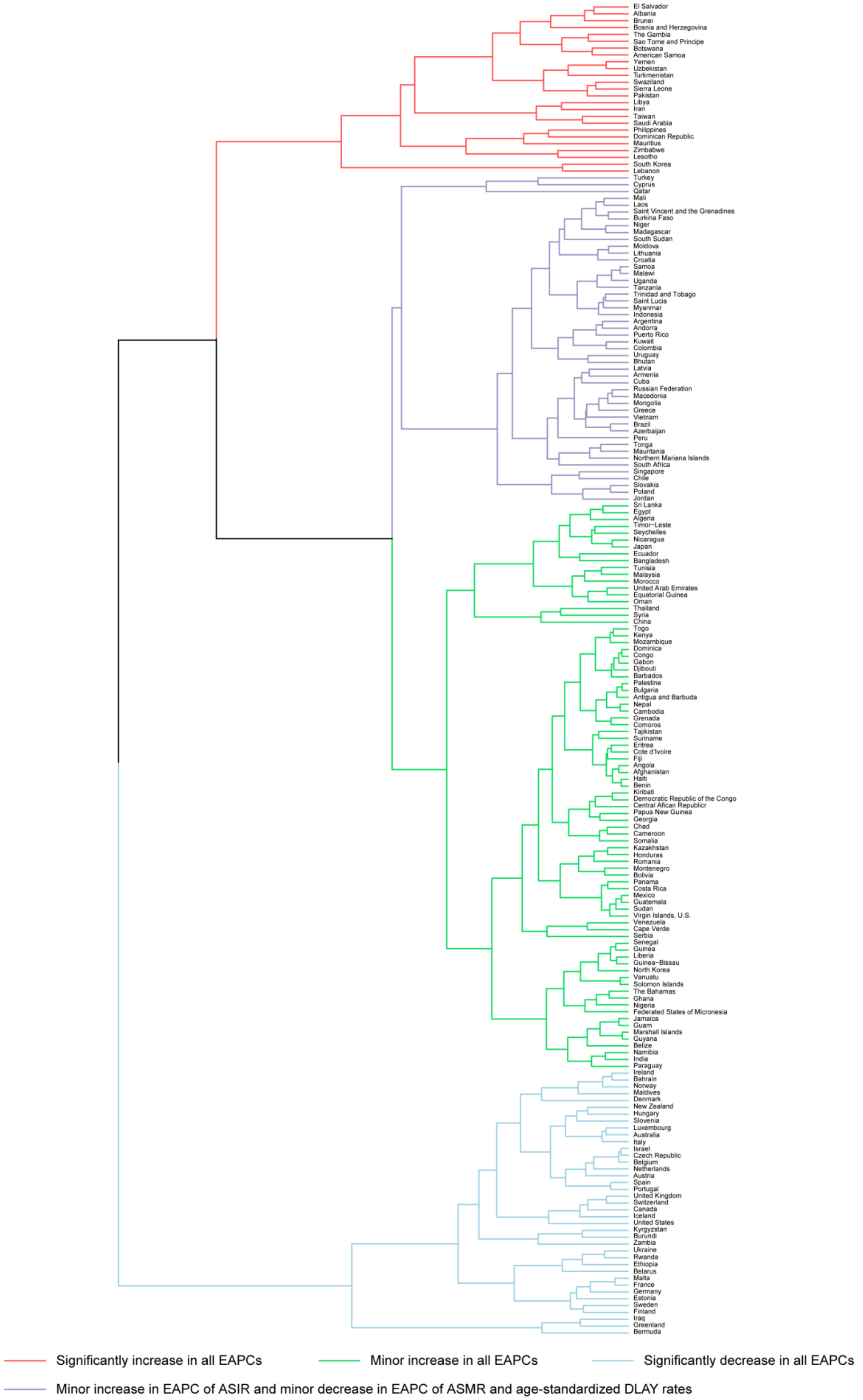
El Salvador
Albania
Brunei
Bosnia and Herzegovina
The Gambia
Sao Tome and Principe
Botswana
American Samoa
Yemen
Uzbekistan
Turkmenistan
Swaziland
Sierra Leone
Pakistan
Libya
Iran
Taiwan
Saudi Arabia
Philippines
Dominican Republic
Mauritius
Zimbabwe
Lesotho
South Korea
Lebanon
Turkey
Cyprus
Qatar
Mali
Laos
Saint Vincent and the Grenadines
Burkina Faso
Niger
Madagascar
South Sudan
Moldova
Lithuania
Croatia
Samoa
Malawi
Uganda
Tanzania
Trinidad and Tobago
Saint Lucia
Myanmar
Indonesia
Argentina
Andorra
Puerto Rico
Kuwait
Colombia
Uruguay
Bhutan
Latvia
Armenia
Cuba
Russian Federation
Macedonia
Mongolia
Greece
Vietnam
Brazil
Azerbaijan
Peru
Tonga
Mauritania
Northern Mariana Islands
South Africa
Singapore
Chile
Slovakia
Poland
Jordan
Sri Lanka
Egypt
Algeria
Timor-Leste
Seychelles
Nicaragua
Japan
Ecuador
Bangladesh
Tunisia
Malaysia
Morocco
United Arab Emirates
Equatorial Guinea
Oman
Thailand
Syria
China
Togo
Kenya
Mozambique
Dominica
Congo
Gabon
Djibouti
Barbados
Palestine
Bulgaria
Antigua and Barbuda
Nepal
Cambodia
Grenada
Comoros
Tajikistan
Suriname
Eritrea
Cote d'Ivoire
Fiji
Angola
Afghanistan
Haiti
Benin
Kiribati
Democratic Republic of the Congo
Central African Republic
Papua New Guinea
Georgia
Chad
Cameroon
Somalia
Kazakhstan
Honduras
Romania
Montenegro
Bolivia
Panama
Costa Rica
Mexico
Guatemala
Sudan
Virgin Islands, U.S.
Venezuela
Cape Verde
Serbia
Senegal
Guinea
Liberia
Guinea-Bissau
North Korea
Vanuatu
Solomon Islands
The Bahamas
Ghana
Nigeria
Federated States of Micronesia
Jamaica
Guam
Marshall Islands
Guyana
Belize
Namibia
India
Paraguay
Ireland
Bahrain
Norway
Maldives
Denmark
New Zealand
Hungary
Slovenia
Luxembourg
Australia
Italy
Israel
Czech Republic
Belgium
Netherlands
Austria
Spain
Portugal
United Kingdom
Switzerland
Canada
Iceland
United States
Kyrgyzstan
Burundi
Zambia
Ukraine
Rwanda
Ethiopia
Belarus
Malta
France
Germany
Estonia
Sweden
Finland
Iraq
Greenland
Bermuda
Significantly increase in all EAPCs
Minor increase in all EAPCs
Significantly decrease in all EAPCs
Minor increase in EAPC of ASIR and minor decrease in EAPC of ASMR and age-standardized DLAY rates

Supplementary Figure 6. The clusters of countries and territories according to the EAPC of breast cancer ASIR, ASMR and age-standardized DALY rates between 1990 and 2017
